# The characterization of Mediator 12 and 13 as conditional positive gene regulators in Arabidopsis

**DOI:** 10.1038/s41467-020-16651-5

**Published:** 2020-06-03

**Authors:** Qikun Liu, Sylvain Bischof, C. Jake Harris, Zhenhui Zhong, Lingyu Zhan, Calvin Nguyen, Andrew Rashoff, William D. Barshop, Fei Sun, Suhua Feng, Magdalena Potok, Javier Gallego-Bartolome, Jixian Zhai, James A. Wohlschlegel, Michael F. Carey, Jeffrey A. Long, Steven E. Jacobsen

**Affiliations:** 1 School of Advanced Agricultural Sciences, Peking University, 100871 Beijing, China; 2 Department of Molecular, Cell and Developmental Biology, University of California at Los Angeles, Los Angeles, CA 90095 USA; 3 Department of Plant and Microbial Biology and Zurich-Basel Plant Science Center, University of Zurich, Zollikerstrasse 107, 8008 Zurich, Switzerland; 4 Basic Forestry and Proteomics Center, Fujian Agriculture and Forestry University, 350002 Fuzhou, China; 5 Department of Biological Chemistry, David Geffen School of Medicine, University of California at Los Angeles, Los Angeles, CA 90095 USA; 6 Institute of Plant and Food Science, Department of Biology, Southern University of Science and Technology, Shenzhen, China; 7 Howard Hughes Medical Institute, University of California at Los Angeles, Los Angeles, CA 90095 USA

**Keywords:** Gene silencing, Chromatin remodelling, Plant molecular biology

## Abstract

Mediator 12 (MED12) and MED13 are components of the Mediator multi-protein complex, that facilitates the initial steps of gene transcription. Here, in an Arabidopsis mutant screen, we identify MED12 and MED13 as positive gene regulators, both of which contribute broadly to *morc1* de-repressed gene expression. Both MED12 and MED13 are preferentially required for the expression of genes depleted in active chromatin marks, a chromatin signature shared with *morc1* re-activated loci. We further discover that MED12 tends to interact with genes that are responsive to environmental stimuli, including light and radiation. We demonstrate that light-induced transient gene expression depends on MED12, and is accompanied by a concomitant increase in MED12 enrichment during induction. In contrast, the steady-state expression level of these genes show little dependence on MED12, suggesting that MED12 is primarily required to aid the expression of genes in transition from less-active to more active states.

## Abstract (web summary)

Mediator is a multiprotein complex required to activate gene transcription by RNAPII. Here, the authors report that MED12 and MED13 are conditional positive regulators that facilitate the expression of genes depleted in active chromatin marks and the induction of gene expression in response to environmental stimuli in Arabidopsis.

## Introduction

In all eukaryotes, the transcription of protein-coding genes is initiated through the formation of the pre-initiation complex (PIC) at transcription start sites (TSSs), involving RNA Polymerase II (Pol II) and multiple general transcriptional factors^1^. PIC assembly, followed by transcriptional elongation, is facilitated by a multicomponent protein complex, termed Mediator, which conveys upstream regulatory information from activators and repressors to the downstream basal transcriptional machinery^2^.

Protein components of Mediator were first identified in *Saccharomyces cerevisiae*^3^ and were later shown to be functionally and structurally conserved in other eukaryotes, including metazoans and plants^4–6^. The core Mediator complex contains 20–30 protein subunits and is organized into three different modules: the head, middle, and tail module^7,8^. In addition, a regulatory module of four protein subunits (Mediator 12 (MED12), Mediator 13, Cyclin-c, and cyclin-dependent kinase 8 (CDK8)), named the CDK8 kinase module, reversibly associates with the core Mediator complex^9,10^. Early studies showed that only a small fraction of the purified core Mediator complex contained the CDK8 kinase module, and Pol II was exclusively present in the fraction that is free of the CDK8 kinase module^9,11–13^. Consistent with this observation, multiple lines of evidence suggest that the CDK8 kinase module acts mainly as a negative regulator during transcription^9,12,14–16^. However, recent results indicate that it also has a positive role in regulating gene transcription^17–21^ and tends to act on a specific group of genes that preferentially respond to environmental and developmental cues^17,18,22–24^. For example, MED12 stabilizes the binding of P300 at hematopoietic stem cell-specific enhancers, which further maintains the active state of these enhancers. In addition, conditional knockout of MED12 resulted in aberrant hematopoiesis in mouse^22^.

Components of the Arabidopsis CDK8 kinase module have also been functionally characterized^4–6^ and were found to have key roles in regulating plant development and defense^6,9,25–31^. Chhun et al. demonstrated that MED13 functions together with VAL1/HSI2 and HDA6 in suppressing the expression of several seed maturation genes through histone deacetylation^31^. In addition, MED13 was shown to form a complex with IAA14 to suppress the transcriptional activity of AUXIN RESPONSE FACTOR 7 (ARF7) and ARF19^30^. Zhu et al. also demonstrated that the Arabidopsis CDK8 kinase module can function as a positive transcription regulator to upregulate key defense genes upon pathogen infection^21^.

Besides transcription factors, eukaryotic gene expression is also strongly influenced by covalent modifications of DNA and histones, which can be either active or repressive. For example, the presence of DNA methylation near TSS is predominantly considered a repressive transcription mark and usually associated with repressive histone modifications, such as H3K9me2^32^. However, it is not uncommon to find actively expressed genes that are DNA methylated in their promoter regions^33^. For instance, in humans it was found that key spermatogenesis regulatory genes are actively transcribed in the presence of promoter DNA methylation^34^. MORC is a GHKL (gyrase, Hsp90, histidine kinase, MutL)-type ATPase-containing protein found in both animal and plant species^35–38^. Mutations of the Arabidopsis MORC proteins reactivate silenced DNA methylated genes, yet have minimal effects on DNA methylation and histone modifications^37,39,40^. Under such circumstance, the composition of the transcriptional machinery and the underlying functional mechanisms are still poorly understood. In this work, we develop a genetic screen in the *morc1* mutant background and identify MED12 and MED13 as conditional positive gene regulators that facilitate the expression of genes depleted in active chromatin marks. In contrast, the steady-state expression of genes that carry high levels of active histone modifications is typically not affected in the *med12* mutant, even though these genes are strongly bound by MED12. We show that MED12 tends to interact with stimulus-responsive genes, and the interaction is dynamically linked to gene expression. MED12 is primarily required to aid the expression of genes in transition from less-active to more-active states.

## Results

### MED12/13 mediates morc1-derepressed *SDC:GFP* expression

**Fig. 1:**
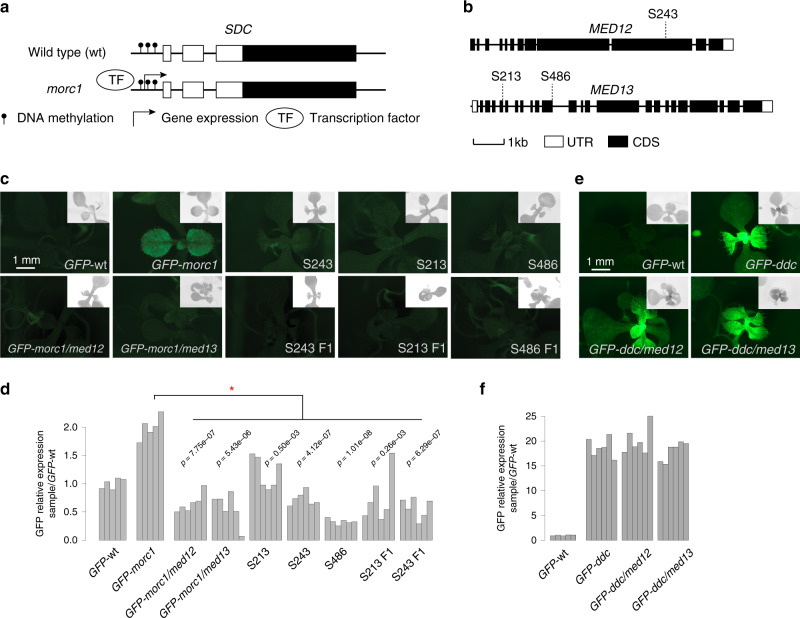
Identification of *med12* and *med13* mutations as *morc1* suppressors. **a** Schematic diagram of *SDC* gene structure and its transcription status in different genetic backgrounds. Open rectangle represents the *SDC* 5’-untranslated region (5’-UTR). Filled rectangle represents the *SDC* coding sequence (CDS). **b** Schematic diagram of the Arabidopsis *MED12* and *MED13* gene structures with the position of each EMS mutation illustrated. Data display conventions as in **a**. **c** GFP fluorescence of plants in different genetic backgrounds. *GFP*-wt and *GFP-morc1* are plants carrying the *SDC:GFP* transgene in wild-type and *morc1* mutant backgrounds, respectively. S243, S213, and S486 are *med12* and *med13* EMS alleles in the *GFP-morc1* background. *GFP-morc1/med12* is the *med12* CRISPR-CAS9 allele re-created in *GFP-morc1* background. *GFP-morc1/med13* is the *med13* T-DNA allele re-created in *GFP-morc1* background. S243-F1 is the F1 resulting from the cross between the CRISPR-CAS9 re-created *GFP-morc1/med12* and S243; S213-F1 and S486-F1 are the F1s resulting from the crosses between the re-created *GFP-morc1/med13* and S213, S486, respectively. Images show 8-day-old seedlings. The auto-fluorescence (gray) from chloroplasts are shown at the top-right corner of each image. **d** Real-time PCR quantification of *SDC:GFP* expression compared between different genetic backgrounds. Each vertical bar represents one biological replicate. A minimum of five biological replicates were analyzed for each mutant background. Red asterisk represents statistically significant difference between the groups under comparison (Student’s *t* test, two-sided). **e** GFP fluorescence of plants in different genetic backgrounds. *GFP*-wt and *GFP-ddc* are plants carrying the *SDC:GFP* transgene in wild-type and *ddc* mutant backgrounds, respectively. *GFP-ddc/med12* mutant is the *med12* CRISPR-CAS9 allele re-created in *GFP-ddc* background. *GFP-ddc/med13* mutant is the *med13* CRISPR-CAS9 allele re-created in *GFP-ddc* background. **f** Real-time PCR quantification of *SDC:GFP* expression compared between different genetic backgrounds. Data display conventions as in **d**. Source data underlying **d**, **f** are provided as a Source data file.

To screen for positive gene regulators that potentially bypass repressive epigenetic marks, we utilized a sensor based on the *SUPRESSOR OF DRM1 DRM2 CMT3* (*SDC*) gene in Arabidopsis. *SDC* is silenced by DNA methylation in wild-type plants and is weakly upregulated in *morc1* mutants, even though the *SDC* promoter DNA remains methylated^37^ (Fig. 1a). Expression of green fluorescent protein (GFP) driven by the endogenous *SDC* promoter, *SDC:GFP*, paralleled that of the endogenous *SDC* gene^37^, arguing that it is a suitable readout for identifying *SDC* regulators. Therefore, ethyl methanesulfonate (EMS) mutations that reduced or abolished *SDC:GFP* expression in the *morc1* background, where DNA methylation and histone modifications remain largely intact, should identify positive gene regulators that act in the presence of repressive chromatin marks. Three alleles (S213, S243, and S486) were identified in the screen (Fig. 1b, Supplementary Fig. 1). S243 was mapped to the gene previously named CENTER CITY (CCT), which is the Arabidopsis homolog of MED12. S213 and S486 were both mapped to the gene named GRAND CENTRAL (GCT)/MACCHI-BOU2 (MAB2), which is the Arabidopsis homolog of MED13^26,41^ (Fig. 1b). Both are single-copy genes in the Arabidopsis genome. All three alleles showed delayed flowering, a phenotype that is typical for *med12* and *med13*^27^ (Supplementary Figs. 2 and 3).

To confirm our mapping results, we re-generated lesions in MED12 and MED13 in the *SDC:GFP-morc1* background. A 2-nucleotide (nt) deletion and frame shift created using CRISPR-CAS9, in the first exon of *MED12*, showed typical *med12* morphological defects as indicated by the delayed flowering time (Supplementary Fig. 2). As expected, *SDC:GFP* is silenced in this mutant (Fig. 1c, d, Supplementary Fig. 4). Crossing a published *med13* null (*gct-2*) into the *SDC:GFP-morc1* background^26^ also silenced *SDC:GFP* (Fig. 1c, d, Supplementary Fig. 4). To confirm that *med12* and *med13* mutations are the causal lesions in each of the EMS mutant lines, we crossed the above re-created mutants with their corresponding EMS mutants. As expected, *SDC:GFP* remained silenced in the F1 of these crosses (Fig. 1c, d, Supplementary Fig. 4). In conclusion, Arabidopsis MED12 and MED13 are required for the expression of the DNA methylated *SDC:GFP* in the *morc1* background.

### **Activation of demethylated*SDC:GFP*** is MED12/13 independent

In *drm1/drm2/cmt3* (*ddc*) mutant, the promoter of *SDC* becomes fully demethylated, which leads to strong activation of *SDC:GFP*^37^. We asked whether MED12 and MED13 are also required for *SDC:GFP* expression in the *ddc* background. Therefore, we generated lesions in *MED12* and *MED13* in the *ddc* mutant carrying the *SDC:GFP* transgene (*SDC:GFP-ddc*) using CRISPR-CAS9. The *SDC:GFP-ddc* mutant carrying a homozygous 1-nt deletion in the second exon of *MED12* was obtained and showed a typical *med12* late flowering phenotype, which indicates a deficiency in MED12 function (Supplementary Figs. 2 and 5). Similarly, a 2-nt deletion in the second exon of *MED13* was introduced into *SDC:GFP-ddc* mutant through CRISPR-CAS9. The resulting plants also showed a typical *med13* late flowering phenotype (Supplementary Figs. 2 and 5). Interestingly, in both cases *SDC:GFP* remained strongly activated and expressed at a level that is comparable to the non-mutagenized *SDC:GFP-ddc* parent (Fig. 1e, f, Supplementary Fig. 4). In conclusion, we showed that MED12 and MED13 are not required for the re-activation of DNA demethylated *SDC:GFP* in the *ddc* background.

### **MED12/13 contribute to*morc1***-mediated gene activation

**Fig. 2:**
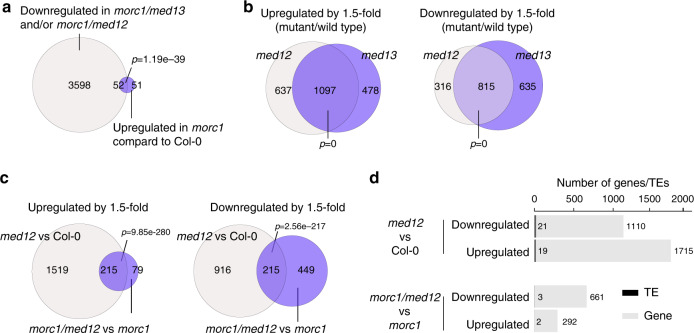
Characterization of *med12/13* differentially regulated genes. **a** Venn diagram showing the overlap between genes that are downregulated in *morc1/med12* and/or *morc1/med13* mutants and those that are upregulated in *morc1* background compared to wild-type (Col-0). Size is proportional to the number of genes defined for each group. *p* value indicates a statistical significance of the overlap (hypergeometric test, one sided). **b** Venn diagram showing the overlap between genes that are upregulated (left) and downregulated (right) in *med12* and *med13* compared to wild-type plants (fold change >1.5, FDR < 0.05). Size is proportional to the number of genes defined for each group. *p* value indicates a statistical significance of the overlap (hypergeometric test, one-sided). **c** Venn diagram showing the overlaps of *med12* upregulated (left) and downregulated (right) genes between wild-type and *morc1* background (fold change >1.5, FDR < 0.05). Data display conventions as in **b**. *p* value indicates a statistical significance of the overlap (hypergeometric test, one sided). **d** Bar chart showing the number of genes (gray) and TEs (black) that are differentially regulated by MED12 in wild-type (upper) and *morc1* (lower) backgrounds. Source data are provided as a Source data file.

A total of 103 genes and transposable elements (TEs), including the *SDC* locus, were upregulated in *morc1* mutants comparing to wild type (Fig. 2a, Supplementary Data 1; >1.5-fold, FDR < 0.05). To ask whether *morc1*-re-activated loci also require MED12 and MED13 for their expression, we compared the transcriptome of *morc1/med12* and *morc1/med13* double mutants with that of *morc1*. The results showed that 52 out the 103 *morc1* upregulated genes/TEs were downregulated when *med12* or *med13* mutation was introduced into the *morc1* background (Fig. 2a, Supplementary Data 1; *p* = 1.19e−39, hypergeometric test), suggesting that a significant proportion of *morc1*-induced transcriptional re-activation requires MED12 and MED13.

To further characterize the function of MED12 and MED13, we performed transcriptome analysis of *med12* and *med13* seedlings to identify MED12 and MED13 regulated genes in the wild-type background. Compared to wild-type plants, 1734 and 1575 genes/TEs were upregulated in *med12* and *med13* mutants, respectively (>1.5-fold, false discovery rate (FDR) < 0.05; Fig. 2b, Supplementary Data 1). 1131 and 1450 genes/TEs were downregulated in *med12* and *med13* mutants, respectively (>1.5-fold, FDR < 0.05; Fig. 2b, Supplementary Data 1). As expected, we observed a significant overlap between *med12* and med*13* differentially regulated genes (DEGs; Fig. 2b), consistent with the notion that MED12 and MED13 function as partners in the same CDK8 kinase module^9,23,24,26,29,42^.

We further compared *med12* DEGs in the wild-type and *morc1* background. We found that there was a significant overlap of *med12* DEGs in the wild-type and *morc1* backgrounds (Fig. 2c). In addition, there was also a large number of genes that were differentially regulated in a background-specific manner (wild type vs. *morc1*; Fig. 2c). The vast majority of *med12* DEGs are categorized as genes but not as TEs regardless of the background origin (wild type vs. *morc1*) (Fig. 2d). Similar results were also obtained when analyzing *med13* DEGs (Supplementary Figs. 6 and 7).

### MED12/13-dependent genes carry unique chromatin signatures

**Fig. 3:**
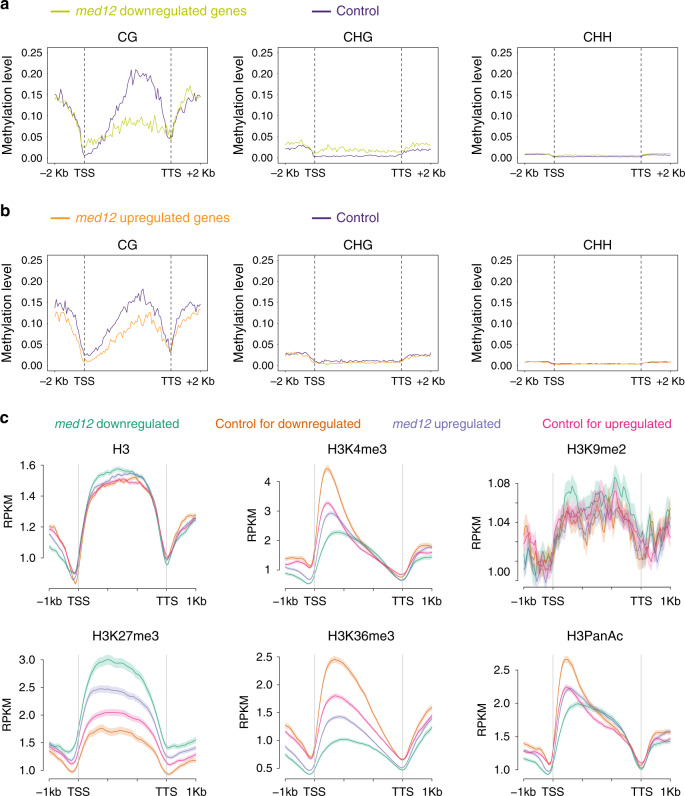
Characterization of epigenetic features of *med12* differentially regulated genes. **a** Average distribution of DNA methylations over *med12* downregulated genes in three different sequence contexts (left to right, CG, CHG, and CHH). Control represents a group of randomly selected MED12 non-DEGs of similar expression levels. −2 kb and +2 kb represent 2 kb upstream of transcription start site (TSS) and 2 kb downstream of transcription termination site (TTS), respectively. **b** Same as in **a**, except for *med12* upregulated genes. **c** Average distribution of histone modifications over *med12* DEGs, distinguishing genes downregulated (green) and upregulated (purple) in *med12* from corresponding controls of similar expression levels (orange and pink, respectively). Top row from left to right, H3, H3K4me3, H3K9me2; Bottom row from left to right, H3K27me3, H3K36me3, H3PanAc. Shaded area represents the standard error (SEM) centered on mean value (dark solid lines). *n* = 3 biologically independent samples.

Given that the DNA methylated *SDC:GFP* in *morc1* mutants showed MED12 and MED13-dependent expression, whereas the promoter de-methylated *SDC:GFP* in *ddc* mutants did not, we asked whether the expressional dependency on MED12 and 13 is associated with the presence of DNA methylation. Therefore, we used the published methylation profile of 10-day seedlings^43^ and quantified the enrichments of CG, CHG, and CHH (H represents A, T, or C) DNA methylation over *med12* and *med13* DEGs (upregulated and downregulated) and compared to that of a set of randomly selected genes with comparable expression levels. The results showed that genes that were downregulated in *med12* and *med13* mutants tended to have higher amounts of CHG DNA methylation in their promoter and gene body regions compared to the randomly selected controls (Fig. 3a, Supplementary Fig. 8, Supplementary Data 2). Interestingly, we also observed a strong depletion of gene body CG methylation for *med12* and *med13* downregulated genes (Fig. 3a, Supplementary Fig. 8). The above trends are either absent or much weaker for *med12* and *med13* upregulated genes (Fig. 3b, Supplementary Fig. 8, and Supplementary Data 2).

We further asked whether certain histone marks were differentially associated with MED12- and MED13-dependent gene in the wild-type background. We profiled genome-wide H3K4me3, H3K9me2, H3K27me3, H3K36me3, and H3PanAc distribution patterns of 8-day-old Col-0 seedlings (same type of tissue used for transcriptome analysis). Interestingly, comparing to a set of control genes of similar expression level, genes that were downregulated in *med12* mutants were strongly depleted of H3K4me3, H3K36me3, and H3PanAc, which are histone marks associated with actively transcribed loci (Fig. 3c). *med12*-downregulated genes are also strongly enriched for the repression-associated H3K27me3 mark (Fig. 3c). Interestingly, *med12*-upregulated genes revealed a similar albeit much weaker trend (Fig. 3c). As expected, no difference in the level of H3K9me2 was observed in any of the comparisons (Fig. 3c), which is perhaps expected given that H3K9me2 is a repressive histone mark typically found over TEs, and the vast majority of *med12* DEGs were protein-coding genes (Fig. 2d, Supplementary Fig. 9). The correlation between MED12 dependency and the depletion of active histone marks was further confirmed in the heatmap of histone chromatin immunoprecipitation and sequencing (ChIP-seq) signals ordered by the expression change of all protein-coding genes in *med12* mutants (Supplementary Fig. 10). Genes that were mis-regulated (both up and down) in *med12* mutants were among the ones that contain the highest levels of H3K27me3 modification and the lowest levels of active histone modifications, including H3K4me3, H3K36me3, and H3PanAc (Supplementary Fig. 10). Similar to what we observed for *med12*, genes downregulated in *med13* mutants were also strongly depleted of active histone marks (H3K4me3, H3K36me3, and H3PanAc) and enriched for repressive histone mark (H3K27me3) (Supplementary Fig. 11). However, it should be noted that even though *med12/13-*downregulated genes were found to contain high levels of H3K27me3, the amount of H3K27me3 marks at these genes are less than that of high-confidence Polycomb repressive complex 2 (PRC2) regulated genes as defined by Xiao et al.^44^ (Supplementary Fig. 12).

We asked whether the chromatin signatures associated with *med12*- and *med13*-downregulated genes were wild-type background specific. Therefore, we profiled H3K4me3, H3K9me2, H3K27me3, H3K36me3, and H3PanAc over *med12* DEGs in the *morc1* background. Similar to what we saw in the wild-type background, *med12*-downregulated genes in the *med12*/*morc1* background were also depleted of all three types of active histone modifications (H3K4me3, H3K36me3, and H3PanAc) and enriched for repressive histone mark, H3K27me3 (Supplementary Fig. 13). This is also true for the *med13*-downregulated genes in the *med13*/*morc1* and *med13*/*ddc* backgrounds (Supplementary Figs. 14 and 15). Together, these results indicate that MED12- and MED13-dependent genes are associated with unique chromatin signatures.

**Fig. 4:**
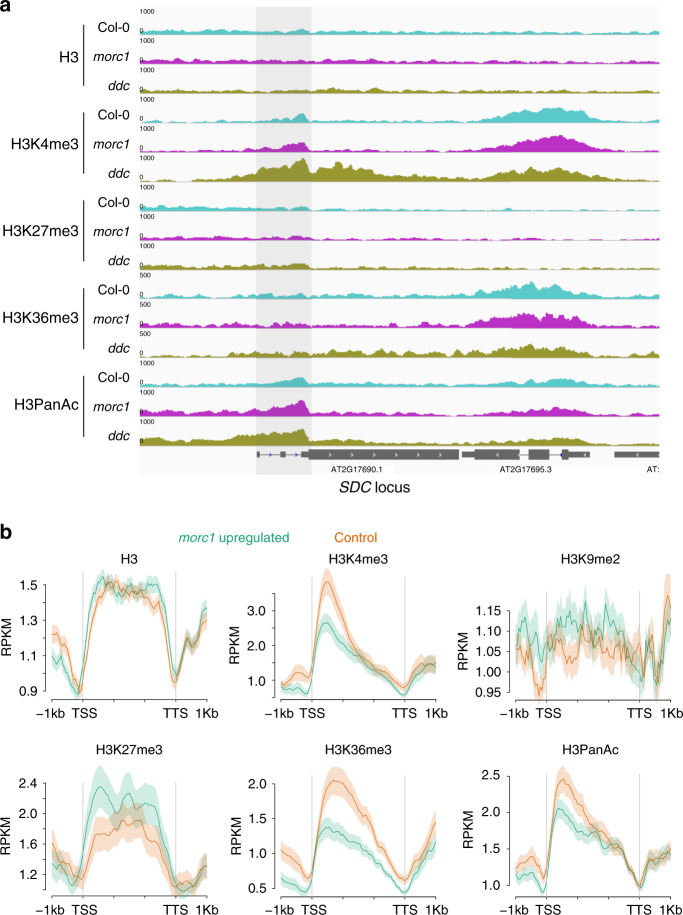
Characterization of the epigenetic features at *SDC* and other *morc1* upregulated loci. **a** Screen shot showing the distribution of (from top to bottom) H3, H3K4me3, H3K27me3, H3K36me3, and H3PanAc over the endogenous *SDC* locus in Col-0 (top track), *morc1* (middle track), and *ddc* (bottom track). Shaded area indicates the region downstream of *SDC* TSS. **b** Average distribution of histone modifications over *morc1* upregulated genes (green) and corresponding controls of similar expression levels (purple). Shaded area represents the standard error (SEM) centered on mean value (dark solid lines). *n* = 3 biologically independent samples.

Given that the expression *SDC:GFP* is only dependent on MED12 and MED13 in the *morc1* background but not in the *ddc* background, we asked whether the loss of MED12/13 dependency in the *ddc* background is also accompanied by a gain of active histone modifications. To test this idea, we examined the enrichments of H3K4me3, H3K36me3, and H3PanAc over the *SDC* locus in the *ddc* background. Compared to wild type, we found a greater than sixfold gain of H3K4me3 downstream of the *SDC* TSS in the *ddc* background but not in the *morc1* background (Fig. 4a, Supplementary Fig. 16). There is also an increased accumulation of H3PanAc in the same region. The comparison did not show visible changes in the accumulation of H3K27me3 and H3K36me3 over the *SDC* locus between different genetic backgrounds (Fig. 4a).

In addition to *SDC*, many other *morc1*-re-activated genes are also downregulated when *med12* and *med13* mutations were introduced into the *morc1* background (Fig. 2a). We asked whether genes that are re-activated in *morc1* also share similar chromatin signatures as those of MED12- and MED13-dependent genes. As expected, when comparing to a set of control genes of similar expression level, *morc1*-re-activated genes also displayed less active histone marks (H3K4me3, H3K36me3, and H3PanAc) and more repressive histone mark (H3K27me3) (Fig. 4b).

### MED12 enrichments positively correlate with gene expression

**Fig. 5:**
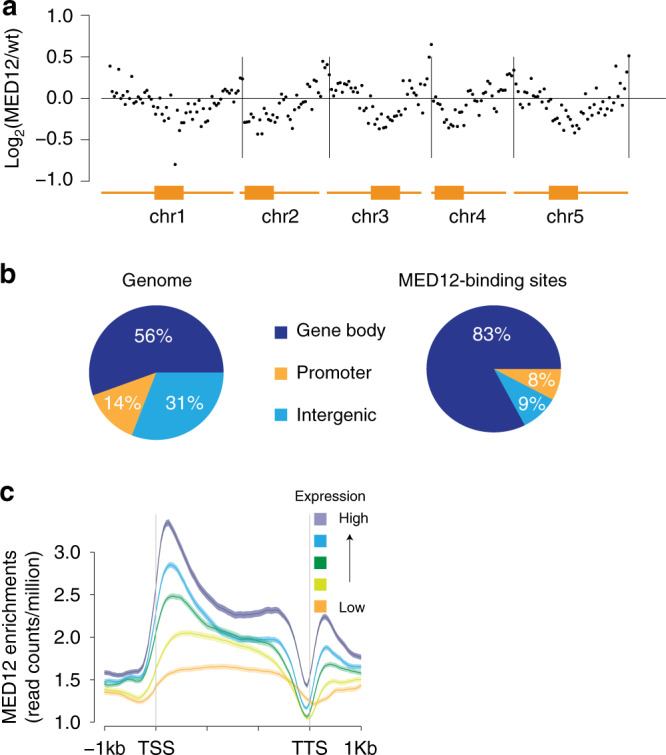
Characterization of MED12-enriched loci. **a** Genome-wide distribution of MED12 on five chromosomes (chr). Each of the five Arabidopsis chromosomes was divided into 500-kb window. The relative enrichments of MED12 within each window was plotted. *Y*-axis represents the log_2_ value of MED12-ChIPseq reads in MED12 complementing plants relative to those in wild-type plants. Orange rectangles indicate the locations of pericentromeric regions. **b** The distribution of MED12 ChIP-seq peaks overlapping with different genomic features (right). The background distribution of different genomic features is shown on the left. **c** Average distribution of MED12 over genes of different expression values. All expressed genes were divided evenly into five groups based on their expression levels in wild-type plants. Orange to yellow to green to blue to purple is low-to-high expression tiers. *Y*-axis represents MED12-FLAG ChIP-seq read counts normalized to sequencing depth. −1 kb and 1 kb represent 1 kb upstream of transcription start site (TSS) and 1 kb downstream of transcription termination site (TTS), respectively.

To further characterize the function of MED12 and MED13, we set out to determine their genome-wide interacting loci. Given that the function of MED12 and MED13 overlap significantly, we chose to focus on MED12 and further introduced a *MED12-YFP-FLAG* transgene driven by the *MED12* endogenous promoter into the *med12* null background. The transgenic plants flowered around the same time as the wild-type control, indicating that the complementation was successful (Supplementary Fig. 2). We then determined MED12 in vivo binding pattern using ChIP-seq and found that MED12 is highly enriched in gene-rich euchromatic regions but depleted in centromeric regions, which are mainly occupied by heterochromatin (Fig. 5a). The majority of MED12 ChIP-seq peaks (Supplementary Data 3) were found inside gene bodies near TSS, showing a strong enrichment there compared to the genome average (Fig. 5b, see “Methods”). It should be noted that MED12 ChIP-seq peaks used for the above analysis were selected based on a highly stringent cut-off (−log_10_*q* value >30). We further divided all Arabidopsis-expressed genes into five groups based on their expression levels (Supplementary Data 4) and examined the enrichment of MED12 within each group. We observed a strong positive correlation between the enrichment of MED12 and the level of gene expression (Fig. 5c).

### MED12 tend to interact with stimuli-response genes

**Fig. 6:**
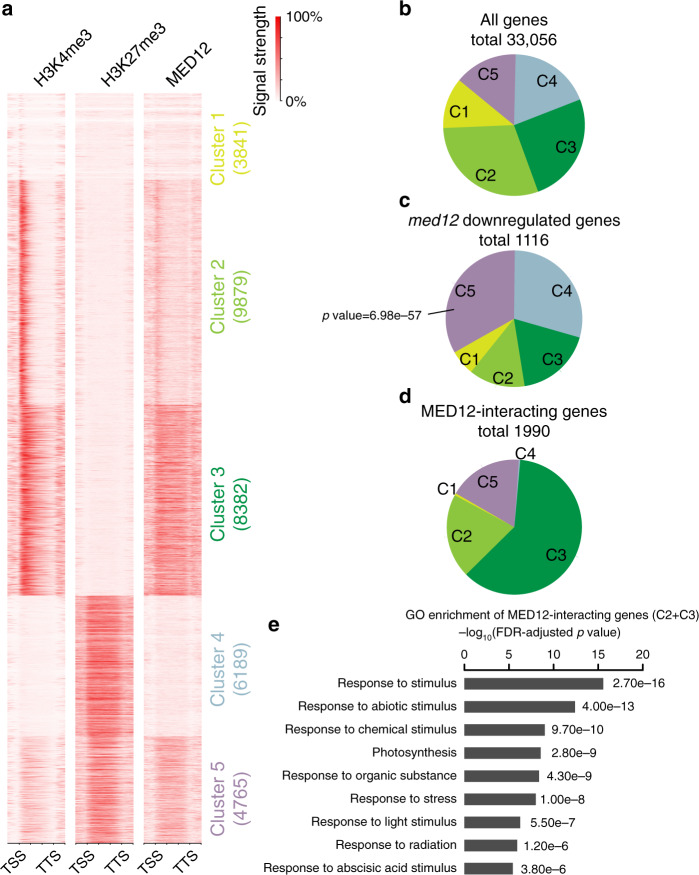
Features of MED12-interacting genes. **a** Heatmap ordered based on *k*-means clustering of H3K4me3, H3K27me3, and MED12 ChIP-seq signals at all Arabidopsis genes (cluster *n* = 5). The number of genes from each cluster is labeled to the right. Color codes are shared with pie chart in **b**–**d**. **b** The percentage of genes derived from each of the five clusters defined in **a**. The percentage was calculated by dividing the number of genes from each cluster by the total number of Arabidopsis genes. **c** The percentage of *med12* downregulated genes derived from each of the five clusters defined in **a**. The percentage was calculated by dividing the number of *med12* downregulated genes from each cluster by the total number of *med12* downregulated genes. *p* value indicates a statistical significance in the enrichment of *med12* downregulated genes in Cluster 5 (hypergeometric test). **d** The percentage of MED12-interacting genes derived from each of the five clusters defined in **a**. Similarly calculated as in **c**, except for MED12-interacting genes. **e** Statistical significance of GO term analysis of MED12-interacting genes derived from Clusters 2 and 3 as defined in **a** (Fisher’s exact test). Source data underlying **b**–**e** are provided as a Source data file.

To obtain a complete view of the chromatin signatures at MED12-interacting loci, we performed *k*-means clustering of all Arabidopsis genes based on the profile of MED12, H3K4me3, and H3K27me3 enrichment. Five gene clusters were obtained from this analysis. We found that MED12-interacting loci are mainly present in three clusters (Fig. 6a): Clusters 2 and 3 carry high levels of H3K4me3 and MED12 but no H3K27me3 enrichment; in contrast, Cluster 5 is characterized by high levels of H3K27me3 and moderate-to-low levels of H3K4me3 and MED12. Importantly, *med12*-downregulated genes are highly enriched for Cluster 5 loci (*p* = 6.98e−57, Fig. 6b vs. Fig. 6c). This further validates our finding that *med12*-downregulated genes are characterized by low levels of H3K4me3 and high levels of H3K27me3. Furthermore, it indicates that the *med12-*downregulated genes are preferentially bound by relatively low levels of MED12.

To assess the effects of MED12 binding, we further define MED12-interacting genes (Supplementary Data 5, see “Methods”). Since we applied a highly stringent cut-off (−log_10_*q* value >30) in defining MED12-interacting genes, genes with only moderate-to-low levels of MED12 interactions were therefore likely to be excluded from this set. We identified 1990 strongly MED12-bound genes, and the majority of them are derived from Clusters 2 and 3 (1620 out of 1990, Fig. 6d), which carry high level of H3K4me3 (Fig. 6a). Interestingly, we observed largely unchanged gene expression levels in wild type vs. *med12* for the vast majority of these strongly MED12-bound genes (Supplementary Fig. 17), indicating that MED12 is largely dispensable for maintaining the steady-state expression at loci that carry high level of H3K4me3. This is also consistent with our early observation that in the *ddc* background H3K4me3 is highly accumulated in the promoter region of *SDC* (Fig. 4a), and the expression *SDC:GFP* is MED12/13 independent in this background (Fig. 1e, f).

Next, we performed gene ontology (GO) term analysis on MED12-bound Cluster 2 and 3 genes (Supplementary Data 6), which found a striking enrichment for stimuli-response genes, with many categories related to light response (Fig. 6e). We reasoned that MED12 may therefore be required for the stimulus-dependent induction of these genes. The accumulation of MED12 over stimulus-response genes may also be dynamic, which increases as genes are de-repressed from the non-induced state. Since many of GO term-defined categories are related to light responses, we decide to test the above hypothesis through light treatments.

### MED12 is required for light-induced gene expression change

**Fig. 7:**
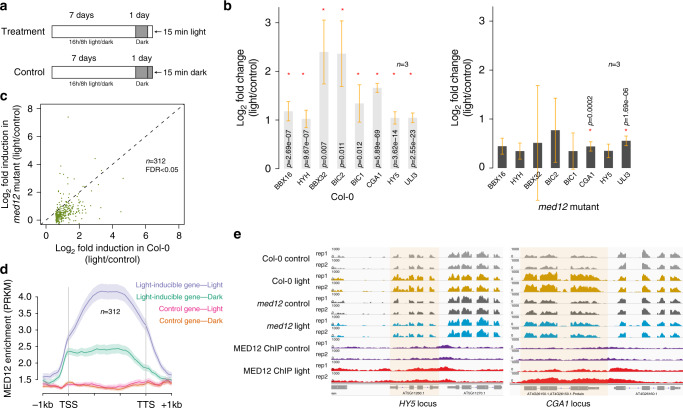
Role of MED12 in mediating light-induced transient gene expression. **a** Schematic illustration of the light and control treatments. **b** Bar plot of light-responsive gene fold induction under light treatments. Left, wild-type plants (Col-0); Right, *med12* mutant plants. Asterisks indicate statistical significance in gene expressional changes (Benjamini–Hochberg adjusted *p* value, two sided). Data are presented as mean values +/− SE. *n* = 3 biologically independent samples. **c** Scatter plot showing the fold induction of light-inducible genes in Col-0 and *med12* mutant plants upon light treatments. Each dot represents one light-inducible gene with its *x*-axis position showing fold induction in Col-0 and *y*-axis position showing fold induction in *med12* mutant. The dotted line represents the 45° reference line. **d** The average distribution of MED12 over light-inducible genes and control genes in the light-treated (purple and red) and non-treated (green and orange) plants. Shaded area represents the standard error (SEM) centered on mean value (dark solid lines). *n* = 3 biologically independent samples. **e** Screen shots showing gene expression and MED12 enrichments at *HY5* (left) and *CGA1* (right) locus under the light-treated and non-treated conditions. Top four tracks, gene expression in Col-0; middle four tracks, gene expression in med*12* mutants; bottom four tracks, MED12 enrichments in MED12 complementing plants. Source data underlying **b**, **c** are provided as a Source data file.

To test whether MED12 is required for light-induced gene expression, both wild-type and *med12* mutant plants were grown under normal light condition for 7 days, followed by a 24-h complete dark treatment. After the dark treatment, plants were brought to light for 15 min prior to sample collection, whereas the control plants were collected in dark without light treatment (Fig. 7a). First, we compared the transcriptome of light-treated and non-treated wild-type plants and identified 312 genes whose expression showed >1.5-fold increase upon the 15-min light treatment (FDR < 0.05; Supplementary Data 1 and 7). Among them, eight genes have been previously studied and were known to regulate plant response to light, which include HY5, HYH, BIC1, BIC2, etc.^45–50^ (Fig. 7b). In contrast, when *med12* mutant plants were subjected to the light treatment, all of these eight genes showed little to no light responsiveness (Fig. 7b), confirming that the light-induced gene expression changes are mediated through MED12. Indeed, the transient light induction of the majority of the 312 light-inducible genes was largely blocked by *med12* mutation (Fig. 7c). In contrast, the steady-state expression levels of these 312 light-inducible genes under normal growth condition are not downregulated in the *med12* mutants (Supplementary Fig. 18). To determine whether the transient light responses were also accompanied by dynamic changes in MED12 binding, we performed MED12 ChIP-seq using MED12-complementing plants under the light-treated and non-treated conditions, annotated MED12-ChIP-seq peaks (Supplementary Data 8), and further defined MED12-interacting genes (Supplementary Data 9). Here we observed a strong increase of MED12 enrichment over the 312 light-inducible genes upon light treatment (Fig. 7d), including HY5 and CGA1, both of which were only found as MED12-interacting genes in the light-treated samples but not the non-treatment controls (Fig. 7e, Supplementary Data 9). In contrast, no increase in MED12 enrichment was observed for similarly expressed non-light-inducible genes (Fig. 7d). Together, the results show that MED12 is required for the transient gene induction in response to light and that MED12 binding is dynamic, displaying increased binding as genes are de-repressed from the non-induced state.

### The profiling of MED12 interactors

To gain mechanistic insights into MED12 action, we identified proteins associated with MED12 by performing immunoaffinity purification followed by mass spectrometry (MS) using MED12-YFP-FLAG expressing plants. Consistent with what is known from yeast and mammals, we identified all three other components of the CDK8 kinase module, which include the Arabidopsis MED13, CDK8, and Cyclin-c (Supplementary Data 10). Furthermore, MED13 ranked highest among all proteins identified based on the number of spectral counts, suggesting that the Arabidopsis MED12 and MED13 likely function closely as partners (Supplementary Data 10). In addition, we also detected many other components comprising the Arabidopsis core Mediator complex (Supplementary Data 10). We did not detect peptides from Pol II, supporting the notion that the CDK8 kinase module and Pol II interact with the core Mediator complex in a mutually exclusive manner^9,11,12^. Furthermore, consistent with the observation that MED12/13 positively regulates *SDC:GFP* expression, we found several members of the Arabidopsis histone acetyltransferase protein families, including AtHAC1, AtHAC5, and AtHAC12 (Supplementary Data 10). AtHAC1 is the Arabidopsis ortholog of animal transcription co-activator P300, which interacts with MED12 to activate gene expression during hematopoiesis in mouse^22^. In summary, our results suggest that MED12, and likely MED13, mainly function as positive gene regulators whose action may involve the recruitment of histone acetyltransferases.

## Discussion

In animals and yeast, protein components of the Mediator CDK8 kinase module have been shown to act as both a positive and a negative regulator for transcription^2^. It has been a challenge to reconcile its contrary roles in gene regulation. In our study, the *med12* and *13* mutations were identified as suppressors of the *morc1*-reactivated *SDC:GFP*. In addition, mutations in *MED12* also blocked the transient gene upregulation upon light treatments (Fig. 7b, c). However, three lines of evidences suggest that MED12 may also play a negative role in regulating the expression of certain genes under particular circumstances. First, genes that are upregulated in *med12* mutants showed higher MED12 enrichment, an effect not observed for *med12*-downregulated genes (Supplementary Fig. 19). Second, 51.7% of *med12*-upregulated genes are directly bound by MED12, whereas 45.6% of *med12*-downregulated genes show direct MED12 binding (Supplementary Fig. 20). Third, the steady-state expression of some light-inducible genes was upregulated in *med12* mutants, although we cannot exclude the possibility of indirect effects (Supplementary Fig. 18).

Our analysis showed that the expression of most strongly MED12 bound genes remained unchanged in *med12* mutants (Supplementary Fig. 17). However, it should be noted that genes of moderate-to-low MED12 interactions may have been excluded from this analysis, because we applied stringent cut-off (−log_10_*q* value >30) in defining MED12-interacting genes (see “Methods”). It is possible that MED12/13 facilitates gene expression when genes are lowly expressed, and only a small amount of MED12/13 are present, which is probably the case for *SDC:GFP* in the *morc1* background. This is consistent with our observation that *med12*-downregulated genes are depleted in active chromatin marks (Supplementary Fig. 10). The scenario may also apply to light-inducible genes upon light treatment, where we observed an increase in gene expression as well as MED12 accumulation (Fig. 7d). We suspect that, as the transcriptional activity increases, MED12 will accumulate at the same time, along with active histone modifications, including H3K4me3 and H3PanAc. Over time, the accumulation of these active histone modifications may finally reach a steady-state level where gene expression is no longer MED12 dependent, such as in the case of *SDC:GFP* in the *ddc* background (Fig. 4a). This can also explain the observation that the steady-state expression of light-inducible genes under normal growth condition was not downregulated in *med12* mutants (Supplementary Fig. 18). Consistent with this, we observed a strong positive correlation between the enrichment of MED12 and the level of gene expression (Fig. 5c). The expression levels of genes strongly bound by MED12 (Supplementary Data 5, see “Methods”) were generally higher compared to the median expression value of all Arabidopsis-expressed genes (Supplementary Fig. 21).

Mechanistically, the enrichment of H3K4me3 may facilitate the recruitment of other transcriptional factors or chromatin remodelers, bypassing a requirement for MED12/13 in transcription^51,52^. High levels of H3K4me3 are a hallmark of high transcriptional activity, where Pol II interacts with the core Mediator complex more frequently during transcription re-initiation, causing less frequent interaction between the core Mediator and the CDK8 kinase module according to the current model^2^. This is often observed in activator-induced transcription^53^. Indeed, both in vivo and in vitro studies have shown that activator-induced transcription is CDK8 kinase module independent^53–56^. Through a re-constituted transcription assay, Sun et al. demonstrated that adding the human MED12-containing kinase module can stimulate the transcription at a basal level but not when the Gal4-VP16 activator was present^57^. The switch of *SDC* from the MED12/13-dependent basal expression in the *morc1* background to the MED12/13-independent highly activated expression in the *ddc* background is consistent with this notion, suggesting that MED12/13 is required to facilitate the expression of genes making the transition from silent to active states.

## Methods

### Plant material

All plant materials used in this study were 8-day old whole seedlings of the Columbia-0 ecotype, unless otherwise described. T-DNA insertion mutants *med12* (*cct-2*, SALK_108241c) and *med13* (*gct-2*, CS65889) were obtained from the Arabidopsis Biological Research Center. The *drm1/drm2/cmt3* triple mutant has been previously described^58^. *SDC:GFP-ddc* was created by transforming the *SDC:GFP* construct into *drm1/drm2/cmt3* triple mutant. The *SDC:GFP* construct has been previously described^37^. *SDC:GFP*-wt and *SDC:GFP-morc1* plants were created by crossing *SDC:GFP-ddc* with Col-0 and *morc1-4* T-DNA mutant (SAIL_1239_C08), respectively. *SDC:GFP*-wt, *SDC:GFP-morc1*, and *SDC:GFP-ddc* all confer glufosinate ammonium resistance delivered by the *SDC:GFP* transgene. Mutation of *MED13* in the *SDC:GFP-morc1* background was created by crossing *gct-2* with *SDC:GFP-morc1*. Mutations of *MED12* in the *SDC:GFP-morc1* and *SDC:GFP-ddc* background and mutation of *MED13* in the *SDC:GFP*-*ddc* background were created using pYao-CAS9 construct developed by Yan et al.^59^. Plants that are homozygous for CAS9-induced mutation with CAS9 transgene segregated away were used for experiments. Guide RNA sequences and genotyping primers are listed in Supplementary Data 11.

### Genetics screening and mapping analysis

Seeds of *SDC:GFP-morc1* were treated with the mutagenic agent EMS. Around 1000 EMS-treated M1 plants were grown and self-propagated to M2 generation. Around 200 seeds of each M2 line were plated on media containing 0.5× Murashige and Skoog basal salt mixture and 25 mg/L glufosinate ammonium. Each M2 seedling was examined for the loss of GFP fluorescence using a Leica MZ16F Fluorescence Stereomicroscope equipped with a GFP Plus filter. In order to map the candidate gene, the GFP-negative M2 of S213, S243, and S486 were backcrossed with the non-mutagenized *SDC:GFP-morc1* parent to generate F1 hybrid, which was further self-propagated to F2 generation. Eight-day-old F2 seedlings grown on media containing 0.5× Murashige and Skoog basal salt mixture and 25 mg/L glufosinate ammonium were examined for GFP fluorescence. Fifty-to-100 GFP-negative F2 individuals of the same F1 progeny were pooled for genomic DNA extraction followed by whole-genome resequencing (Kapa Hyper Prep Kit, Kapa Biosystems). Mapping by sequencing were performed using SHOREmap with default settings^60^. Specifically, raw reads were first mapped to Arabidopsis TAIR10 reference genome [https://www.arabidopsis.org/download/index-auto.jsp?dir=%2Fdownload_files%2FGenes%2FTAIR10_genome_release%2FTAIR10_chromosome_files]. The resulting SAM files were converted to BAM files and further sorted using samtools sort function (v1.2)^61^. The resulting sorted BAM files were further converted into.vcf files using the samtools mpileup function and bcftools call -mv function. The resulting.vcf files were further converted into SHOREmap marker file using the SHOREmap convert --marker function. To map for the causal allele, we used SHOREmap backcross function with the following parameters: -plot-bc –marker-score 40 –marker-freq 0.0 –min-coverage 10 –max-coverage 80 –bg-cov 1 –bg-freq 0.0 –bg-score 1 -non-EMS –cluster 1 –marker-hit 1 -verbose. The mapped causal alleles within specified genomic regions were further annotated using SHOREmap annotate function.

### RNA-seq library preparation and transcriptome analysis

Total RNA was isolated using TRI-zol (Direct-zol, ZYMO RESEARCH) from 8-day -old whole seedlings grown on MS plates supplemented with 1% sucrose under long-day conditions. All plants were grown together at the same time under the same condition and harvested at the same time. Around 1 μg of total RNA was used as input for RNA-seq library preparation following the standard protocol of TruSeq Stranded mRNA Library Prep Kit (Illumina). Fifteen cycles of amplification were used to generate the cDNA libraries. The libraries with concentration adjusted to 10 nmol/L were sequenced on HiSeq2000 or HiSeq4000 platform to generate 50-bp single-end reads. Reads were quality filtered using trim_galore (v0.5.0), Babraham Bioinformatics [http://www.bioinformatics.babraham.ac.uk/projects/trim_galore/] with the following parameters: --fastqc --phred64 --stringency 3, and all other parameters default. The filtered reads were aligned to Arabidopsis TAIR10 reference genome using STAR (v2.7.0c)^62^ with the following parameters: --alignSoftClipAtReferenceEnds No –outFilterType BySJout –outFilterMismatchNmax 999 –outSAMtype BAM SortedByCoordinate –outFilterIntronMotifs RemoveNoncanonical. A java program MarkDuplicates.jar from the picard-tools suite was used to remove PCR duplicates from the resulting BAM files. We then obtained read counts of all genes and TEs according to Araport11 annotation file in the .gtf format using htseq-count v.0.6.1.p1^63^ with the following parameters: --format=bam --stranded=reverse --idattr=gene_id --mode=union. We then loaded the count data for all samples into DESeq2^64^. To identify DEGs, a cut-off value of >1.5-fold change and adjusted *p* value >0.05 were applied. All other parameters were of default value. GEO accession: GSE143835.

### DNA methylation analysis

The whole-genome bisulfite sequencing data of 10-day-old Col-0 seedlings (GSM958801–SRR520367) was retrieved from Zhong et al.^43^. Raw bisulfite sequencing reads were mapped to TAIR10 reference genome using BSMAP (v2.90)^65^ with the following parameters: -v 2 -w 1. Reads that contain three or more consecutive CHH sites were considered as non-converted reads and have been removed. The enrichments of DNA methylation over *med12* and *med13* DEGs were plotted using the ViewBS MethOverRegion function of the ViewBS package at default settings^66^.

### ChIP and sequencing

Histone modification ChIP-seq was performed using 10 g of 8-day-old seedlings grown on MS plates supplemented with 1% sucrose under long-day conditions^67^. Five μL anti-H3 antibody (Abcam #1791), 5 μL of anti-H3K4m3 antibody (Millipore 04-745), 5 μL of anti-H3K9me2 antibody (Abcam #1220), 10 μL of anti-H3K27me3 antibody (Millipore 07-449), 5 μL of anti-H3K36me3 antibody (Abcam #9050), and 5 μL of anti-H3PanAc antibody (Active Motif #39139) were used for each ChIP sample. All antibodies used in this study were undiluted. Specifically, samples were first vacuum infiltrated for 10 min in 1× phosphate-buffered saline (PBS) buffer containing 1.5 mM EGS, followed by three washes using 1× PBS buffer. Crosslinking was stopped by 1× PBS buffer containing  0.125 M glycine. Tissues were flash frozen in liquid nitrogen followed by homogenization using a RETCH homogenizers (30 Hz for 1 min). The resulting fine powder was resuspended with Nuclear Isolation Buffer (50 mM Hepes, 1 M Sucrose, 5 mM KCl, 5 mM MgCl_2_, 0.6% Triton X-100, 0.4 mM PMSF, 5 mM Benzamidine, 1× protease inhibitor cocktail tablet (Roche, 14696200)). Tissue debris were removed by filtering through Miracloth, followed by centrifugation for 20 min at 2880 × *g* at 4 °C. The pellet was resuspended in 1 mL Extraction buffer 2 (0.25 M sucrose, 10 mM Tris-HCl pH8, 10 mM MgCl_2_, 1 % Triton X-100, 5 mM BME, 0.1 mM PMSF, 5 mM Benzamidine, and 1× protease inhibitor cocktail tablet), followed by centrifugation for 10 min at 12,000 × *g* at 4 °C. The pellet was then resuspended in 500 μL Extraction buffer 3 (1.7 M sucrose, 10 mM Tris-HCl pH8, 2 mM MgCl_2_, 0.15% Triton X-100, 5 mM BME, 0.1 mM PMSF, 5 mM Benzamidine, 1× protease inhibitor cocktail tablet). The resuspended solution was layered over an equal volume of fresh Extraction buffer 3, followed by centrifugation for 1 h at 12,000 × *g* at 4 °C. The pellet was then resuspended in 400 μL ice cooled Nuclei Lysis Buffer (50 mM Tris-HCl pH8, 10 mM EDTA, 1% SDS, 0.1 mM PMSF, 5 mM Benzamidine, 1× protease inhibitor cocktail tablet). In all, 1.7 mL ChIP Dilution Buffer (1.1% Triton X-100, 1.2 mM EDTA, 16.7 mM Trish-HCl pH8, 167 mM NaCl, 0.1 mM PMSF, 5 mM Benzamidine, 1× protease inhibitor cocktail tablet) was added to the nuclei lysate followed by chromatin shearing on Bioruptor Plus (Diagenode) with the following settings: 30 s ON/30 s OFF, Max power, 17 cycles. The quality of sheared chromatin was checked by running on a 2% agarose gel. If successful, 100 μL was saved as input, with the rest split into desired amount of aliquots and incubated with different antibodies (5 μL of anti-FLAG antibody, Sigma-A8592-1MG) overnight with rotation at 4 °C. After overnight incubation, 25 μL protein A and 25 μL protein G Dynabeads (Invitrogen 10004D/10002D) was added to the solution, followed by additional incubation at 4 °C for 2 h. The magnetic beads were sequentially washed twice with 1 mL of low salt buffer (150 mM NaCl, 0.2% SDS, 0.5% Triton X-100, 2 mM EDTA, 20 mM Tris-HCl pH8), 1 mL of high salt buffer (200 mM NaCl, 0.2% SDS, 0.5% Triton X-100, 2 mM EDTA, 20 mM Tris-HCl pH8), 1 mL of LiCl wash buffer (250 mM LiCl, 1% Igepal, 1% sodium deoxycholate, 1 mM EDTA, 10 mM Tris-HCl pH8), and 1 mL of TE buffer (10 mM Tris-HCl pH8, 1 mM EDTA). Each wash step was taken at 4 °C for 5 min with rotation. The immunocomplex was eluted from the beads twice with 250 μL elution buffer (1% SDS, 10 mM EDTA, 0.1 M NaHCO_3_) by incubating at 65 °C for 2 h. The reverse-crosslink was done by adding 20 μL 5 M NaCl and incubation at 65 °C overnight. DNA was purified by Protease K treatment followed by phenol/chloroform/isoamyl alcohol extraction and ethanol precipitation. Libraries were prepared using the Ovation Ultralow System V2 (NuGEN #0344) and sequenced on HiSeq2000/4000 platform to generate 50-bp single-end reads. Reads were quality filtered using trim_galore, Babraham Bioinformatics, [http://www.bioinformatics.babraham.ac.uk/projects/trim_galore/] with the following parameters: --fastqc --phred64 --stringency 3 and all other parameters default. The filtered reads were aligned to Arabidopsis TAIR10 reference genome using STAR^62^ with the following parameters: --alignSoftClipAtReferenceEnds No –outFilterType BySJout –outFilterMismatchNmax 999 –outSAMtype BAM SortedByCoordinate –outFilterIntronMotifs RemoveNoncanonical. A java program MarkDuplicates.jar from the picard-tools suite (Picard Toolkit. 2019. Broad Institute, GitHub Repository; http://broadinstitute.github.io/picard/) was used to remove PCR duplicates from the resulting BAM files. To define ChIP-seq peaks, BAM files were loaded into macs2 (v2.1.2)^68^ callpeak function with the following parameters: -f BAM -nomodel -g 1.3e8 -B -q 0.01. Peaks obtained from the above procedures were further filtered using the cut-off value −log_10_*q* value >30. To visualize the enrichments of different histone modifications over *med12* and *med13* DEGs, we used ngsplot (v2.61) with default settings^69^. The heatmaps showing gene clusters based on the pattern of histone modifications were generated using the ngs.plot *k*-means clustering algorithms with default settings. GEO accession: GSE143835.

### Analysis of MED12 genome-wide distribution

Each of the five Arabidopsis chromosomes was divided into 500-kb window. The genomic coordinates of the 500-kb windows in the bed file format were generated using the bedtools makewindows function (-w 500000)^70^. The resulting bed file and the bam file obtained from MED12-3xFLAG ChIP-seq were both loaded into the samtools bedcov function to generate the coverage file, where the total read base counts for each 500-kb window region were reported. The above procedures were performed for two biological replicates of MED12-3xFLAG ChIP-seq data and one replicate of Col-0 ChIP-seq data. The signals from MED12-3xFLAG ChIP-seq were further normalized to that of the Col-0 control. Finally, the log_2_ averaged values of the two biological replicates were plotted in R.

### Analysis of the genomic features at MED12-binding sites

We used two different methods to define the overlaps between MED12 ChIP-seq peaks and different genomic features (gene body, promoter, intergenic). In the first method, we require that each feature has to occupy >50% region of a given MED12 ChIP-seq peak (−log10*q* value >30) to be counted. In this case, the bed file containing genomic coordinates of start and stop sites of each MED12-ChIPseq peak were loaded into the bedtools intersect function. The following parameter was provided in addition to the default settings: -f 0.501. The total length in nts of ChIP-seq peaks (−log10*q* value >30) overlapping with the same genomic feature was used to calculate the relative ratio of each feature. The average ratio of two MED12 ChIP-seq biological replicates were taken and plotted. In the second method, a feature will be counted if the summit of a given MED12 ChIP-seq peak falls in the region of that feature. In this case, the bed files containing the genomic coordinates of each MED12 ChIP-seq peak summit was loaded into the bedtools intersect function without providing additional parameters. Both methods showed similar results. Figure 5b was drawn using method one.

### Characterization of MED12-interacting genes

To define MED12 strongly bound genes, the genomic coordinates of gene regulatory regions were obtained by extending 300 bp upstream and 500 bp downstream from TSS of each gene. MED12 ChIP-seq peaks were first filtered using a cut-off of −log_10_*q* value >30. Peaks passing the cut-off (Supplementary Data 3, sheet 1) were used to intersect with the 800-bp regulatory window of each gene. A gene is defined as a strong MED12-interacting gene if there is a >40% overlap between the 800-bp regulatory window and the MED12 ChIP-seq peaks. Two biological replicates of MED12 ChIP-seq data were analyzed using the above method. Results from the two replicates were further combined with duplicates removed.

To examine the overlap between MED12-interacting genes and *med12* DEGs, the genomic coordinates of *med12* DEGs were obtained by extending from transcription termination site to 500 bp upstream of TSS of each gene. All MED12 ChIP-seq peaks (Supplementary Data 3, sheet 2) that were generated by MACS2 with the default settings were used to intersect with the genomic region of each gene. A *med12* DEG is defined as a MED12-interacting gene if there is a >40% reciprocal overlap between the gene under consideration and the MED12 ChIP-seq peaks. Two biological replicates of MED12 ChIP-seq data were analyzed using the above method. Results from the two replicates were further combined with duplicates removed. Genes that interact with MED12 in the dark and light conditions were defined using the same criteria as for MED12-interacting *med12* DEGs.

### **Affinity** purification and MS

Immunoprecipitation (IP) followed by MS was done according to Moissiard et al.^71^ with the following modifications. Ten grams of flowers were ground in liquid nitrogen and resuspended in 40 mL ice-cold IP buffer [50 mM Tris·HCl pH 8.0, 150 mM NaCl, 5 mM MgCl2, 0.1% Nonidet P-40, 10% (vol/vol) glycerol, 1× Protease Inhibitor Mixture (Roche)], dounced two times using a glass homogenizer, and centrifuged for 10 min at 4 °C at 20,000 × *g*. Two hundred μL M2 magnetic FLAG-beads (Sigma M8823) were added to the supernatant and incubated for 120 min rotating at 4 °C. M2 magnetic FLAG-beads were washed five times in ice-cold IP buffer for 5 min rotating at 4 °C, and immunoprecipitated proteins were eluted two times with 300 μL 250 μg/mL 3×-FLAG peptides (Sigma F4799) in TBS (50 mM Tris-HCl pH 7.4, 150 mM NaCl) for 15 min at 25 °C. The elution was precipitated by trichloroacetic acid, washed two times in ice-cold acetone, and subjected to MS analyses.

### Reporting summary

Further information on research design is available in the Nature Research Reporting Summary linked to this article.

## Supplementary information

Supplementary Information

Reporting Summary

Description of Additional Supplementary Files

Supplementary Dataset 1

Supplementary Dataset 2

Supplementary Dataset 3

Supplementary Dataset 4

Supplementary Dataset 5

Supplementary Dataset 6

Supplementary Dataset 7

Supplementary Dataset 8

Supplementary Dataset 9

Supplementary Dataset 10

Supplementary Dataset 11

## Data Availability

Data supporting the findings of this work are available within the paper and its Supplementary Information files. A reporting summary for this article is available as a Supplementary Information file. The datasets generated and analyzed during the current study are available from the corresponding author upon request. ChIP-sequencing data (fastq files and bigwig files) and RNA-sequencing data (fastq files and DESeq2 count files) obtained during the current study have been deposited in NCBI GEO Datasets with the accession code GSE143835. The source data underlying Figs. 1d, f, 2, 1b–e, and 7b, c, as well as Supplementary Figs. 2, 7, 16–18, 20, and 21 are provided as a Source data file. Source data are provided with this paper.

## Source data

Source Data
