# Supplementary material for: The characterization of Mediator 12 and 13 as conditional positive gene regulators in Arabidopsis
Source: Nat Commun. 2020 Jun 3;11:2798. doi: 10.1038/s41467-020-16651-5 (PMC7271234; doi:10.1038/s41467-020-16651-5)
Supplement: Supplementary file 1 — Supplementary Information [file 41467_2020_16651_MOESM1_ESM.pdf]

The characterization of Mediator 12 and 13 as conditional positive gene regulators in *Arabidopsis*

Liu *et al*.

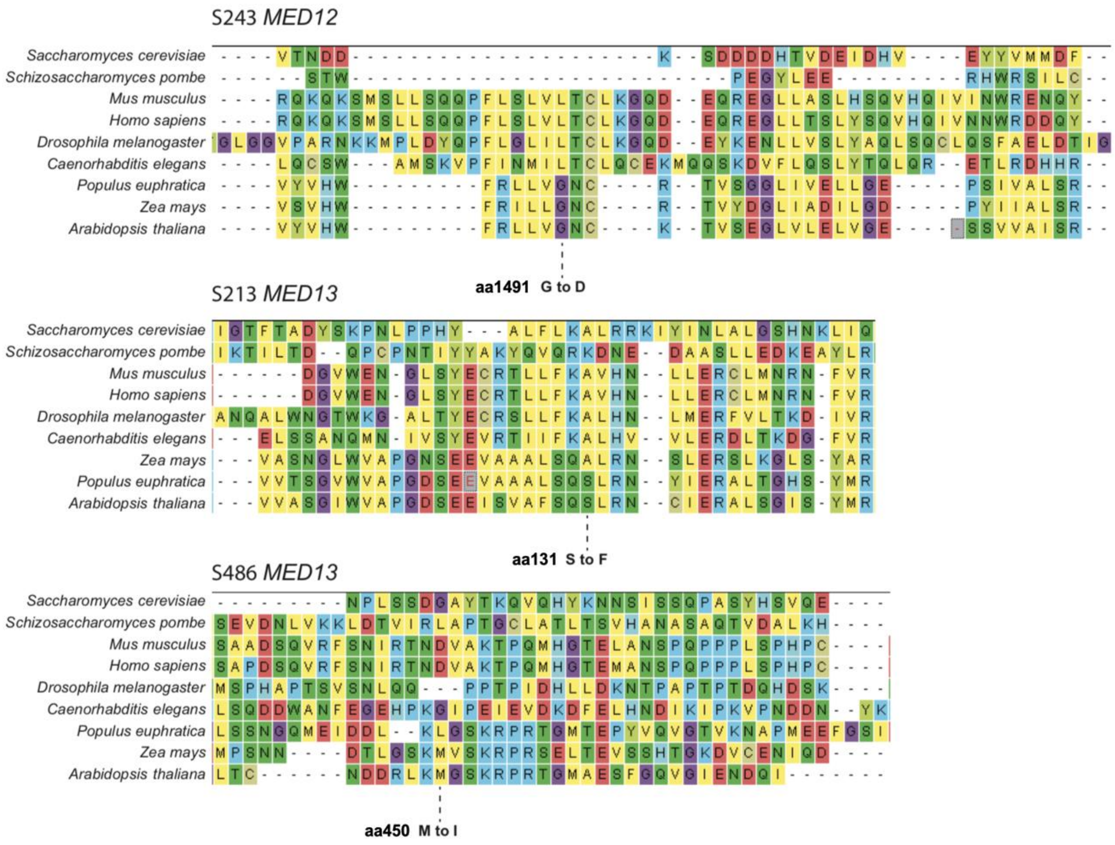

Supplementary Figure 1. Alignments of MED12 and MED13 protein sequences from different model organisms. The alignment was created using MEGA7 with default settings[1]. Amino acids of similar chemical properties were colored the same. The dotted line indicates the position of lesion observed in each EMS mutant.

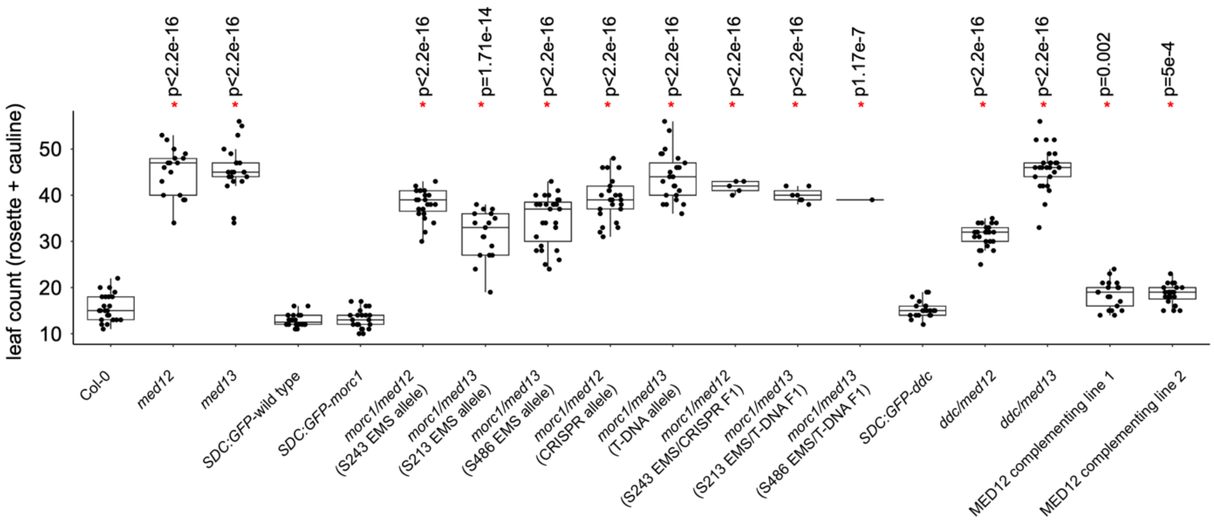

Supplementary Figure 2. Flowering time of plants in different genetic backgrounds. Boxplots show the median total number of leaves (rosette + cauline) when plants are flowering (thick line), extent of the 1st and 3rd quartile range (box), values extending to 1.5 times the interquartile range (whiskers). Dots represent individual data points. Red asterisks indicate statistically significant difference when compared to Col-0 ($p<0.05$, Student's *t*-test, two-sided). n ≥15 biologically independent plant individuals. Source data are provided as Source Data file.

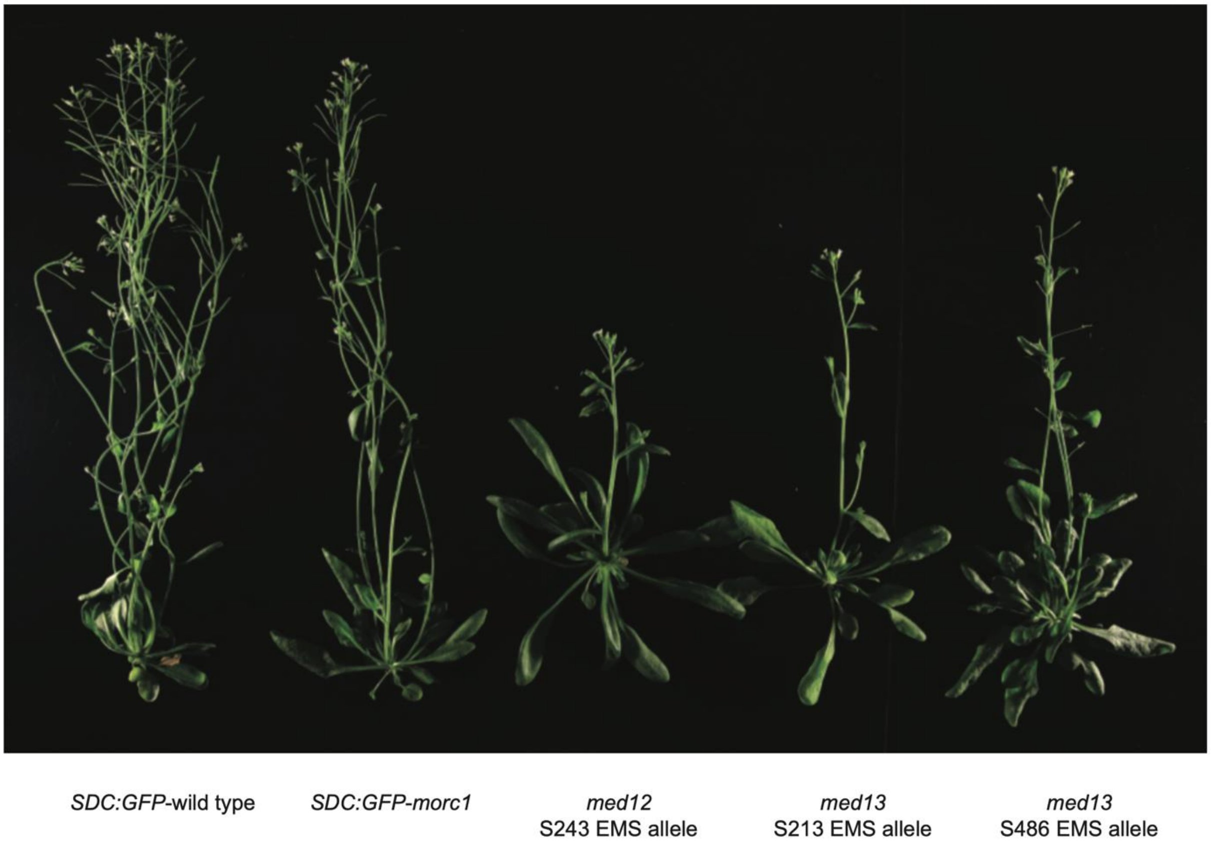

Supplementary Figure 3. Phenotype of the representative *med12* and *med13* EMS mutants. Picture was taken for 10-week old adult plants. 10-20 biological replicates were examined for each mutants and controls. Pictures of one representative plant from each group were taken. From left to right, *SDC:GFP*-wild type, *SDC:GFP-morc1*, EMS mutant S243 (*med12*), EMS mutant S213 (*med13*), and EMS mutant S486 (*med13*).

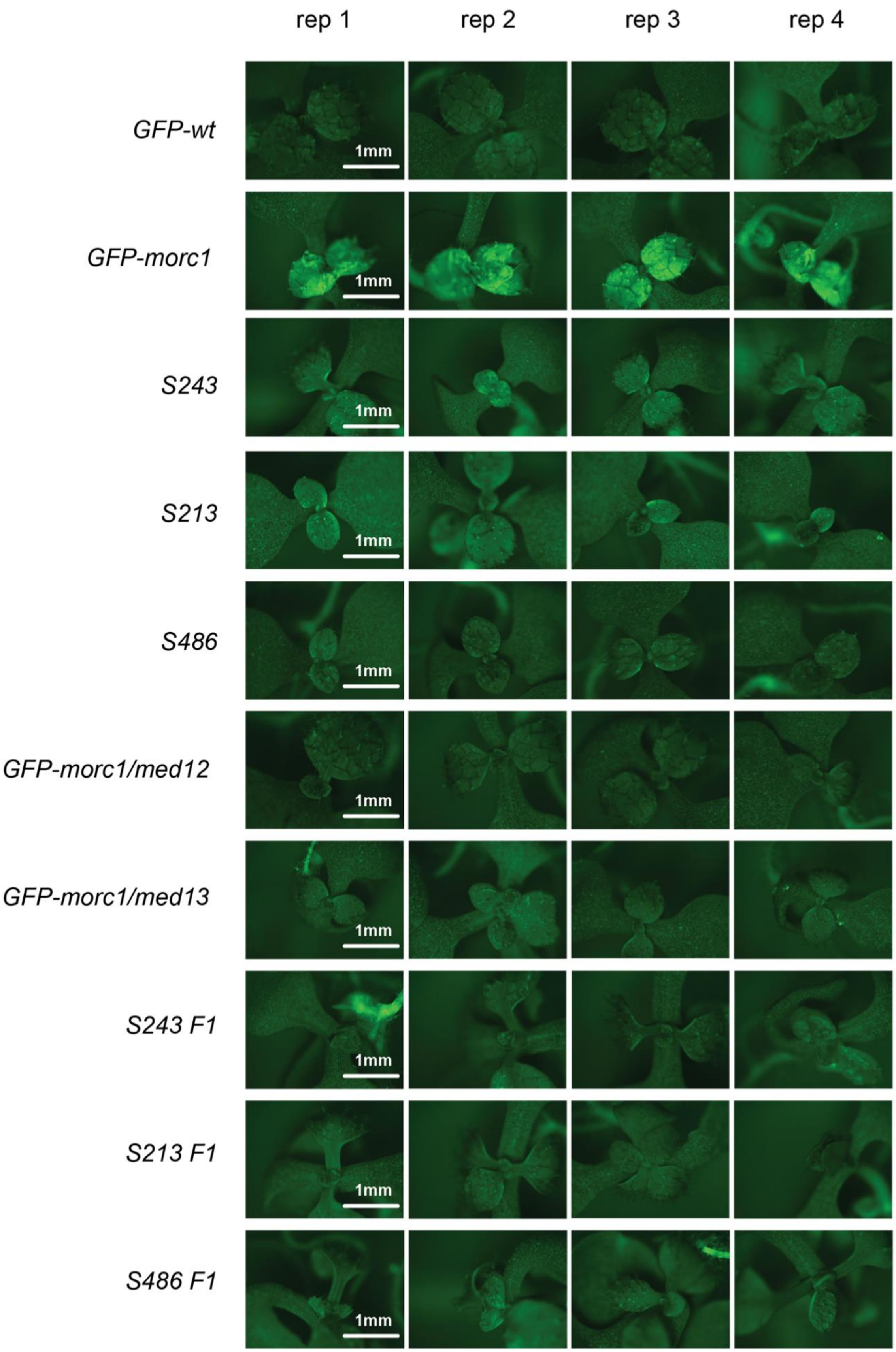
rep 1
rep 2
rep 3
rep 4
GFP-wt
GFP-morc1
S243
S213
S486
GFP-morc1/med12
GFP-morc1/med13
S243 F1
S213 F1
S486 F1
1mm

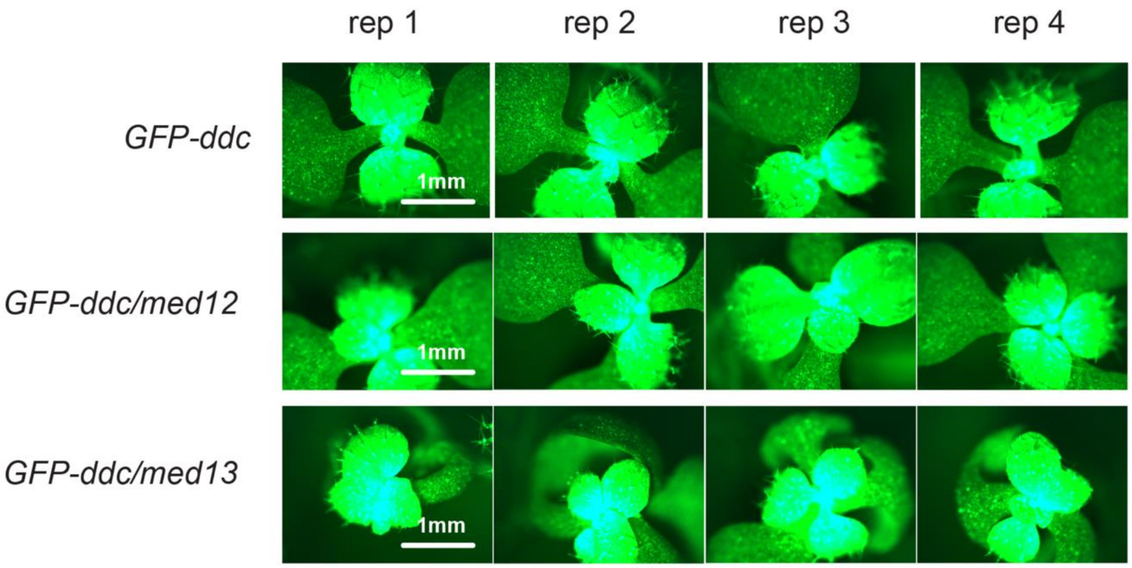

Supplementary Figure 4. GFP fluorescence of 8-day old seedlings in different genetic backgrounds. Four more biological replicates from each genetic background shown in Fig. 1c and 1e are displayed. Data display conventions as in Fig. 1c and 1e. The experiment was repeated independently for four times with similar results.

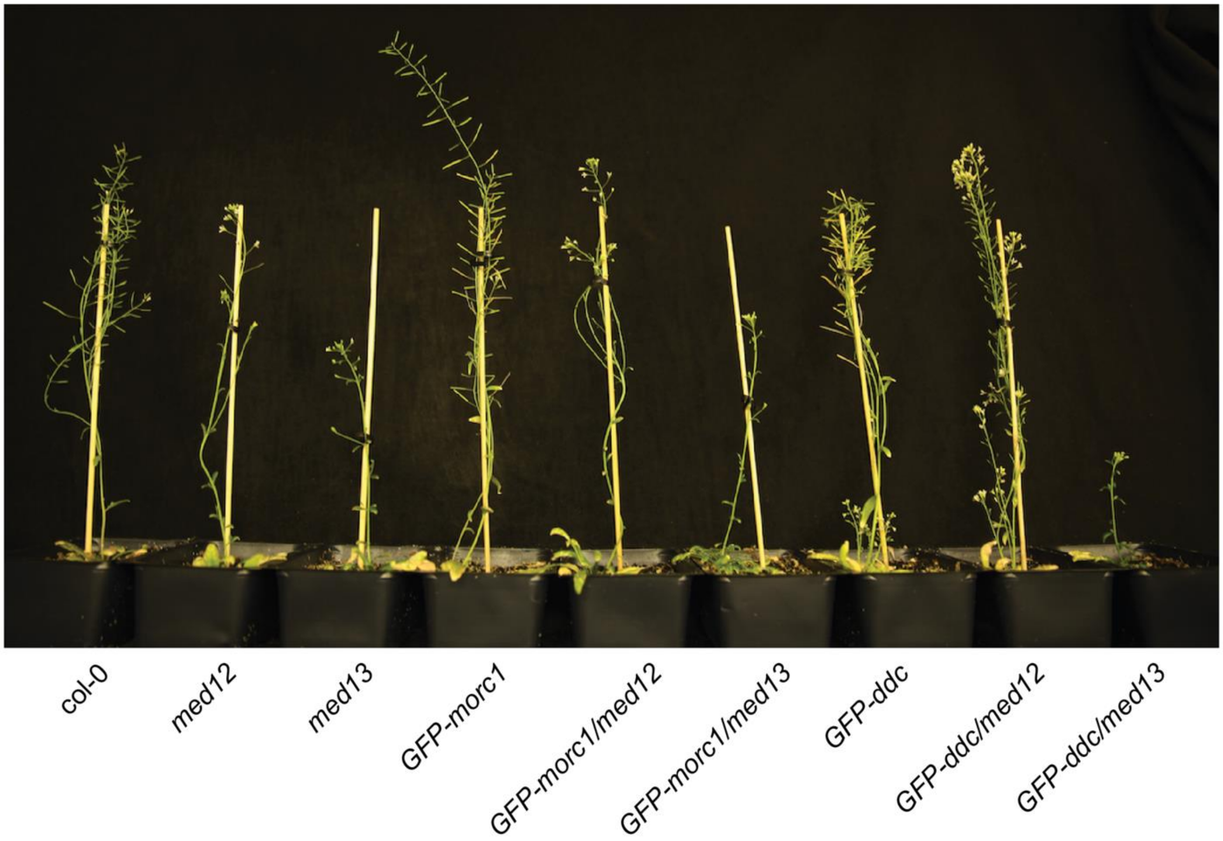

Supplementary Figure 5. Phenotype of the representative re-created *med12* and *med13* mutants in the *morc1* and *ddc* backgrounds. Picture was taken for 10-week old adult plants. All plants were grown at the same time under the same growth condition. 10-20 biological replicates were examined for each mutants and controls. Pictures of one representative plant from each group were taken. From left to right, Col-0, *med12* mutant (*cct-2*, SALK_108241c), *med13* mutant (*gct-2*, CS65889), *SDC:GFP-morc1*, *SDC:GFP-morc1/med12* (CRISPR-CAS9 allele), *SDC:GFP-morc1/med13* (*gct-2* allele), *SDC:GFP-ddc*, *SDC:GFP-ddc/med12* (CRISPR-CAS9 allele), *SDC:GFP-ddc/med13* (CRISPR-CAS9 allele).

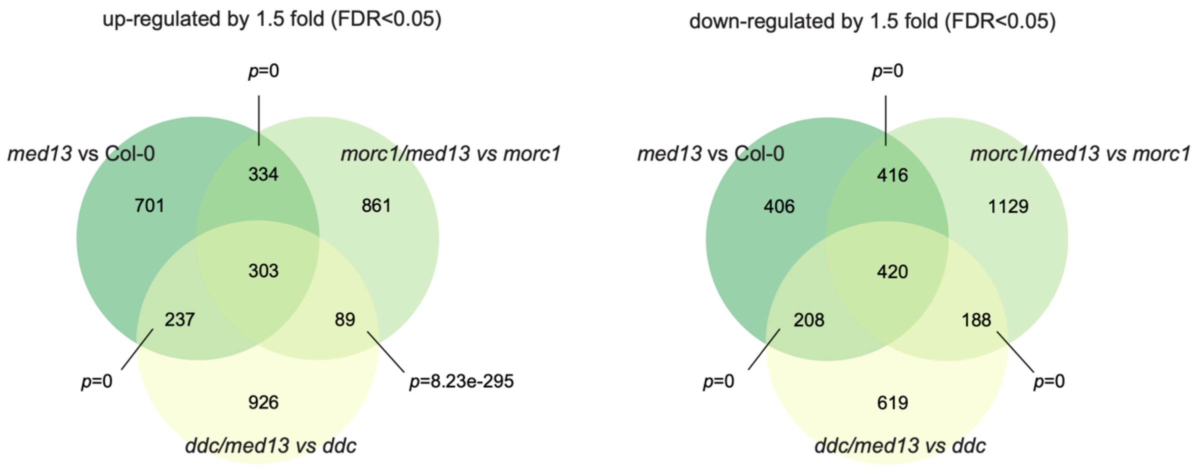

Supplementary Figure 6. Venn diagram showing the overlaps of *med13* up- (left) and down-regulated (right) genes between wild-type, *morc1*, and *ddc* backgrounds (fold change >1.5, FDR <0.05). *p* value indicates a statistical significance of the overlap (hypergeometric test, one-sided).

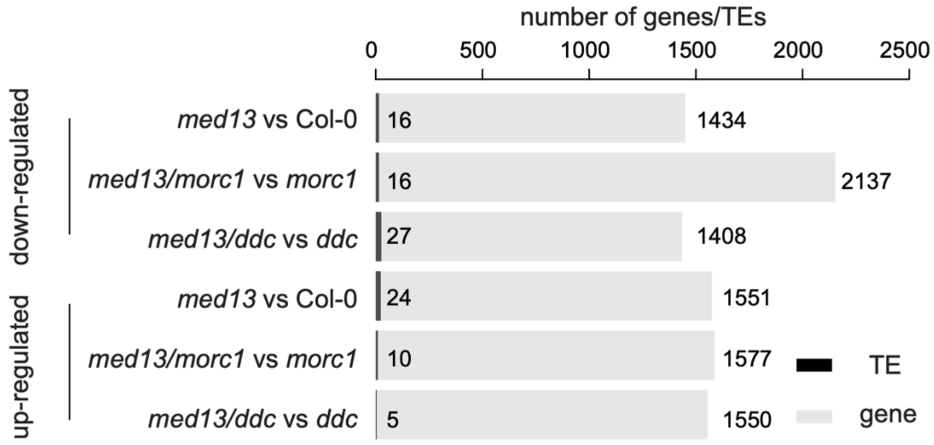

Supplementary Figure 7. Bar-chart showing the number of genes (gray) and TEs (black) that are differentially regulated by MED13 in wild-type (upper), *morc1* (middle), and *ddc* (bottom) backgrounds. Source data are provided as Source Data file.

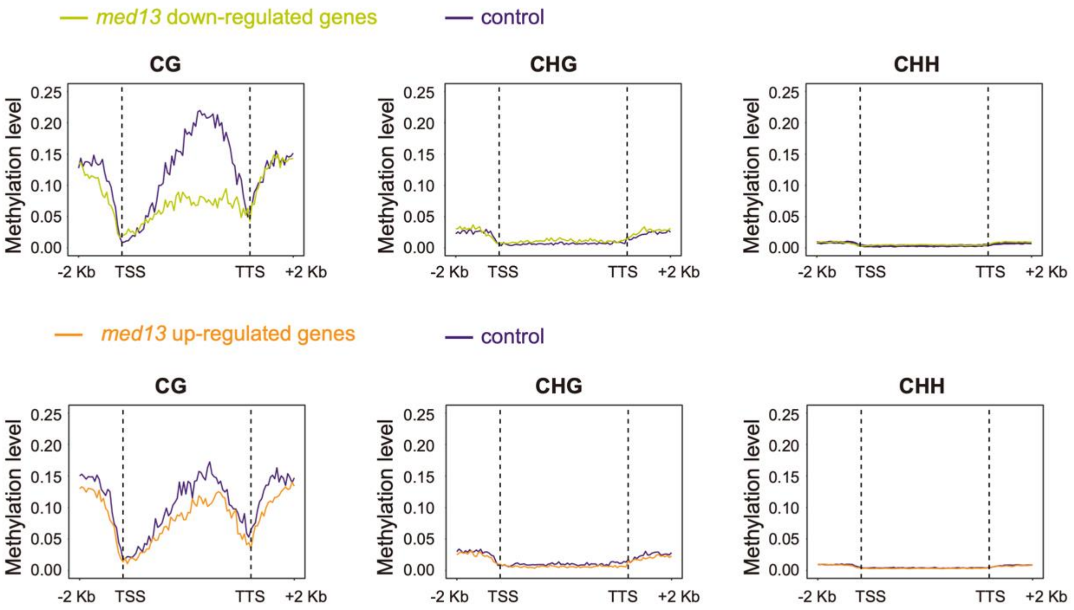

Supplementary Figure 8. Average distribution of DNA methylations over *med13* down- (top) and up-regulated (bottom) genes in three different sequence contexts (left to right, CG, CHG, and CHH). Control represents a group of randomly selected MED13 non-DEGs of similar expression levels.

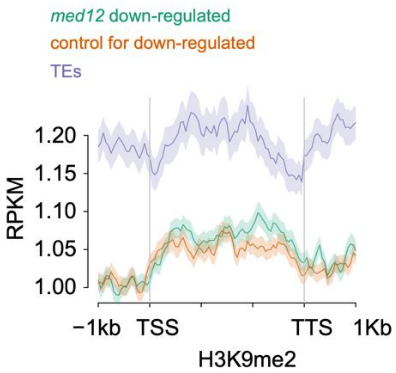

Supplementary Figure 9. Average distribution of H3K9me2 histone modifications over *med12* down-regulated genes (green) and a group of randomly selected TEs (purple). Control represents a group of randomly selected MED12 non-DEGs of similar expression levels (orange). Shaded area represents the standard error (SEM) centered on mean value (dark solid lines). n=3 biologically independent samples.

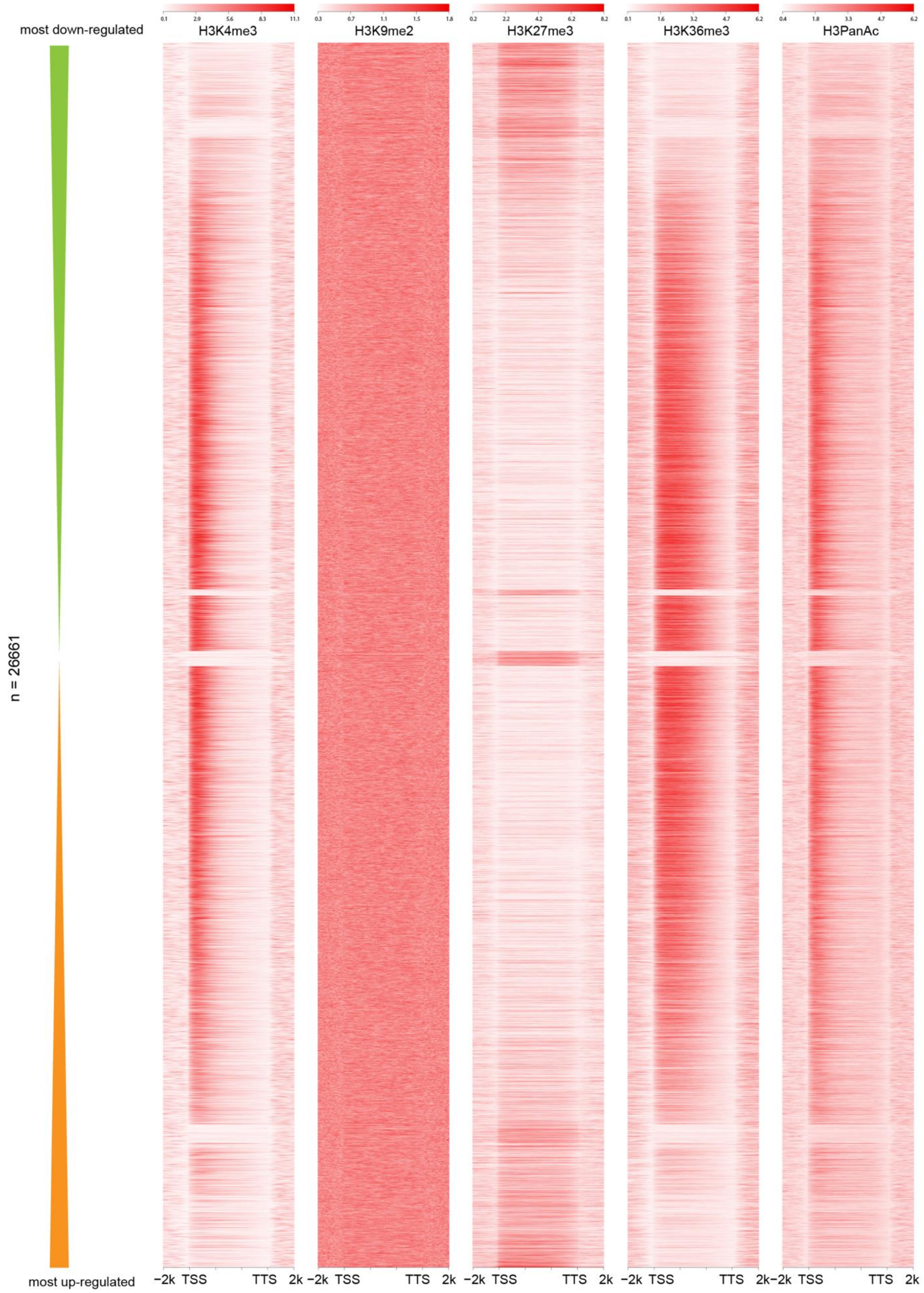
most down-regulated
0.1
2.9
5.6
8.3
11.1
H3K4me3
0.3
0.7
1.1
1.5
1.8
H3K9me2
0.2
2.2
4.2
6.2
8.2
H3K27me3
0.1
1.6
3.2
4.7
6.2
H3K36me3
0.4
1.8
3.3
4.7
6.2
H3PanAc
n = 26661
most up-regulated
−2k TSS TTS 2k
−2k TSS TTS 2k
−2k TSS TTS 2k
−2k TSS TTS 2k
−2k TSS TTS 2k

Supplementary Figure 10. Heatmaps of histone ChIP-seq signals over Arabidopsis genes/TEs. Genes and TEs were ranked (from top to bottom) by fold reduction in *med12* mutants compared to wild-type plants. Left to right, H3K4me3, H3K9me2, H3K27me3, H3K36me3, and H3PanAc.

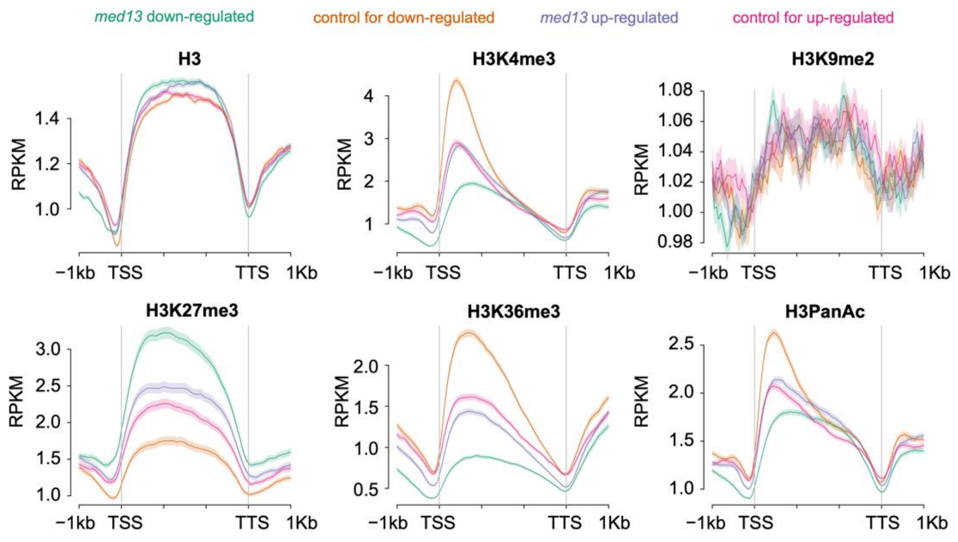

Supplementary Figure 11. Average distribution of histone modifications over *med13* DEGs in wild-type background, distinguishing genes down- (green) and up-regulated (purple) in *med13* from corresponding controls of similar expression levels (orange and pink, respectively). Top row from left to right, H3, H3K4me3, H3K9me2; Bottom row from left to right, H3K27me3, H3K36me3, H3PanAc. Shaded area represents the standard error (SEM) centered on mean value (dark solid lines). n=3 biologically independent samples.

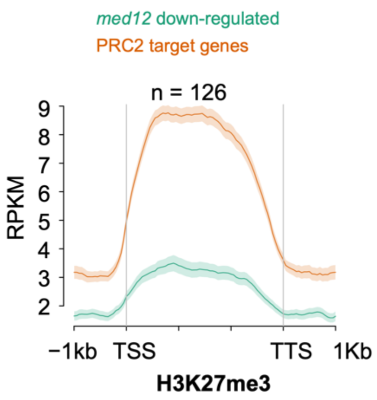

Supplementary Figure 12. Average distribution of H3K27me3 histone modifications, distinguishing classic PRC2 target genes (orange) and *med12* down-regulated genes (green). The list of PRC2 target genes (n=126) were obtained from Xiao *et al*.[2] The top 126 *med12* most down-regulated genes were used for comparison. Shaded area represents the standard error (SEM) centered on mean value (dark solid lines). n=3 biologically independent samples.

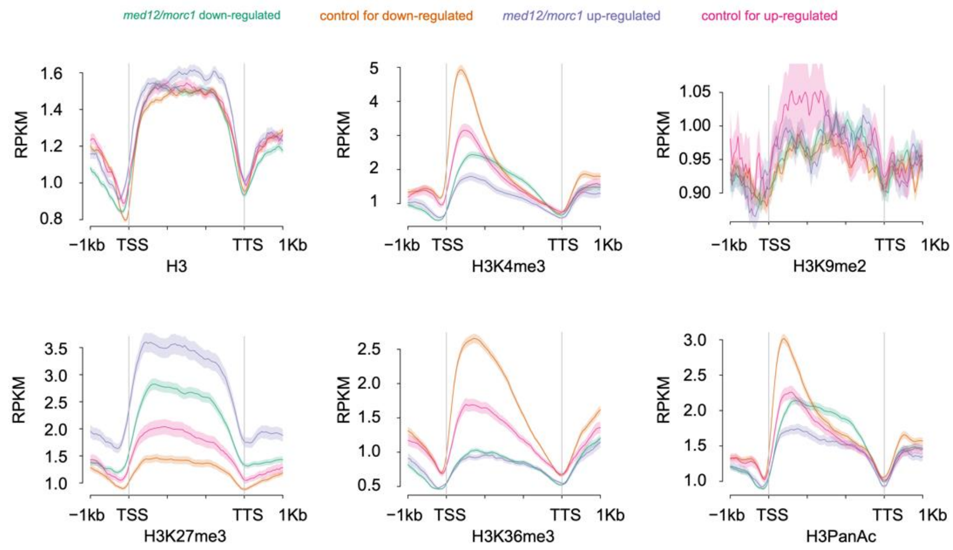

Supplementary Figure 13. Average distribution of histone modifications over *med12* DEGs in *med12*/*morc1* background, distinguishing genes down- (green) and up-regulated (purple) in *med12*/*morc1* from corresponding controls of similar expression levels (orange and pink, respectively). Top row from left to right, H3, H3K4me3, H3K9me2; Bottom row from left to right, H3K27me3, H3K36me3, H3PanAc. Shaded area represents the standard error (SEM) centered on mean value (dark solid lines). n=3 biologically independent samples.

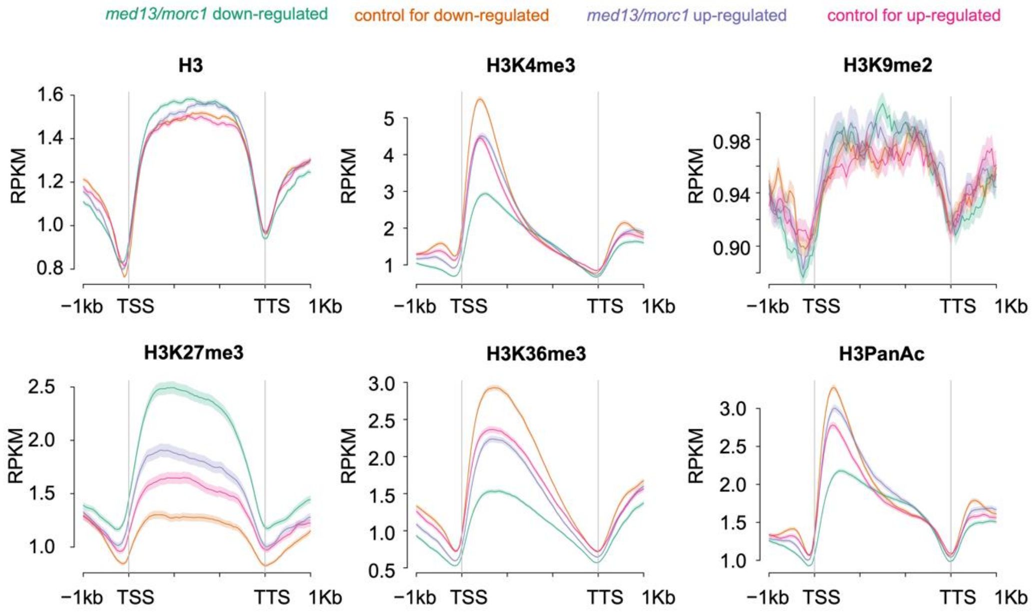

Supplementary Figure 14. Average distribution of histone modifications over *med13* DEGs in *med13/morc1* background, distinguishing genes down- (green) and up-regulated (purple) in *med13/morc1* from corresponding controls of similar expression levels (orange and pink, respectively). Top row from left to right, H3, H3K4me3, H3K9me2; Bottom row from left to right, H3K27me3, H3K36me3, H3PanAc. Shaded area represents the standard error (SEM) centered on mean value (dark solid lines). n=3 biologically independent samples.

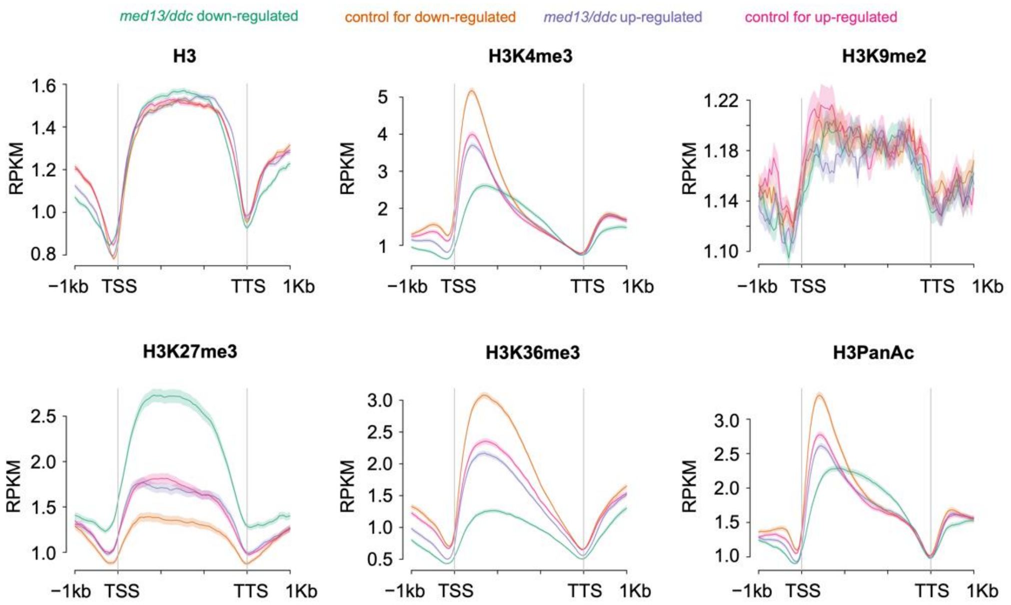

Supplementary Figure 15. Average distribution of histone modifications over *med13* DEGs in *med13*/*ddc* background, distinguishing genes down- (green) and up-regulated (purple) in *med13*/*ddc* from corresponding controls of similar expression levels (orange and pink, respectively). Top row from left to right, H3, H3K4me3, H3K9me2; Bottom row from left to right, H3K27me3, H3K36me3, H3PanAc. Shaded area represents the standard error (SEM) centered on mean value (dark solid lines). n=3 biologically independent samples.

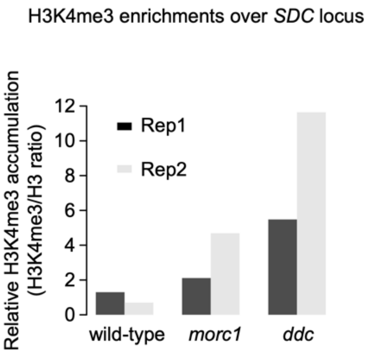

Supplementary Figure 16. Quantification of H3K4me3 enrichments over the endogenous *SDC* locus in wild-type (left), *morc1* (middle), and *ddc* (right) backgrounds. Results from two biological replicates were quantified in each case. Each bar represents the calculation from one biological replicate. The total amount of H3K4me3 ChIP-seq reads that were mapped to *SDC* locus were normalized to that of H3 ChIP-seq reads from the same region. Source data are provided as Source Data file.

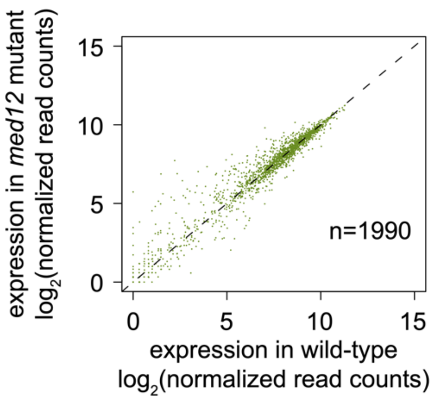

Supplementary Figure 17. Scatter-plot showing the steady-state expression levels of strongly MED12-interacting genes in wild-type and *med12* mutants. Each dot represents one of the 1,990 MED12-interacting genes (*see method*). X-axis, log_2 gene expression level in wild-type. Y-axis, log_2 gene expression level in *med12* mutants. The gene expression level in normalized read counts was directly obtained from the DEseq2 base_mean value, which is the sequencing depth normalized read numbers. The dotted line represents the 45° reference line. Source data are provided as Source Data file.

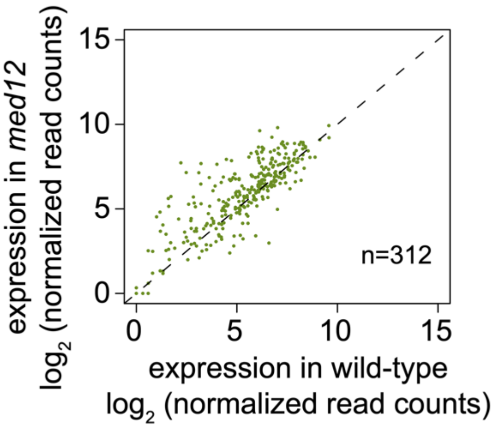

Supplementary Figure 18. Scatter-plot showing the steady-state expression levels of light-inducible genes in wild-type and *med12* mutants. Each dot represents one of the 312 light-inducible genes identified in the wild-type background (*see method*). X-axis, $\log_2$ gene expression level in wild-type. Y-axis, $\log_2$ gene expression level in *med12* mutants. The gene expression level in normalized read counts was directly obtained from the DEseq2 base_mean value, which is the sequencing depth normalized read numbers. The dotted line represents the 45° reference line. Source data are provided as Source Data file.

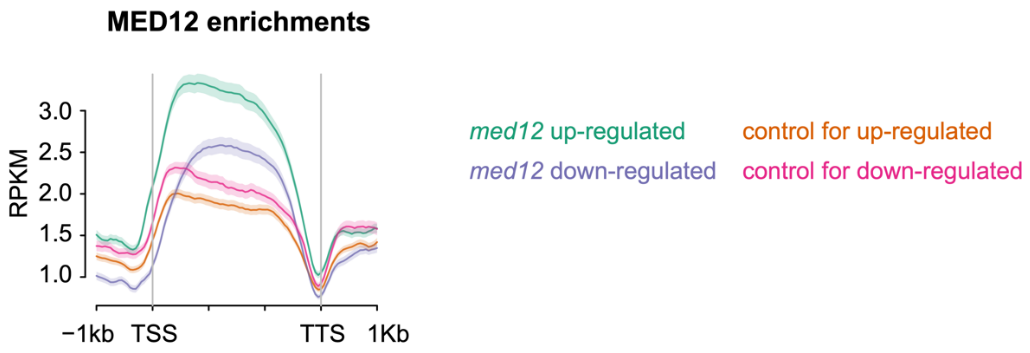

Supplementary Figure 19. Average distribution of MED12 over *med12* DEGs in wild-type background, distinguishing genes up- (green) and down-regulated (purple) in *med12* from corresponding controls of similar expression levels (orange and pink, respectively). Shaded area represents the standard error (SEM) centered on mean value (dark solid lines). n=3 biologically independent samples.

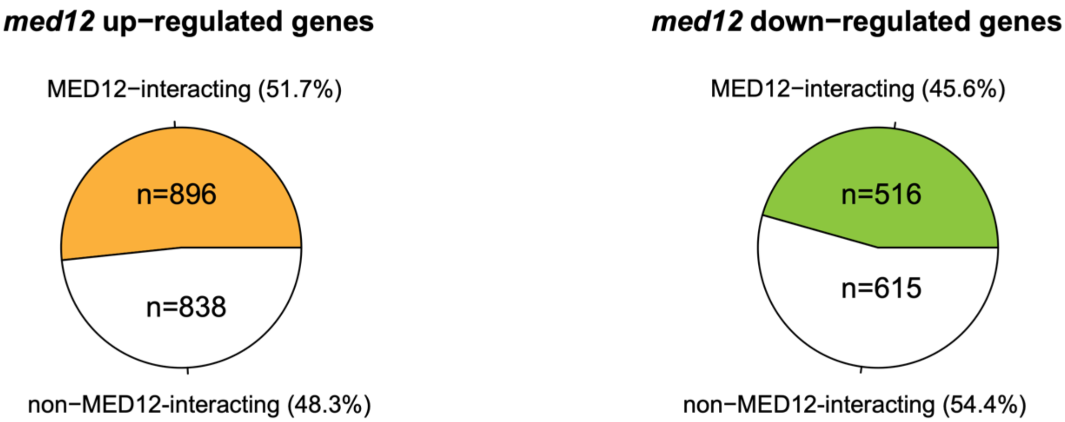

Supplementary Figure 20. The percentage of *med12* differentially regulated genes that are directly bound by MED12. Left, *med12* up-regulated genes. Right, *med12* down-regulated genes. Source data are provided as Source Data file.

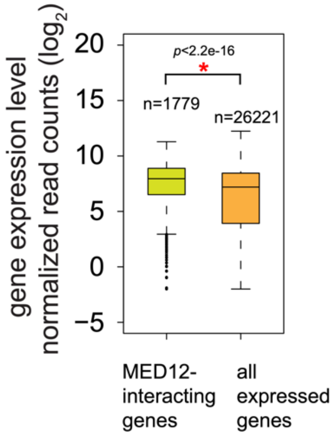

Supplementary Figure 21. The expression of MED12-interacting genes and all expressed genes. Boxplots show the median $\log_2$ value of the normalized read counts (thick line, the base_mean value of DEseq2), extent of the 1st and 3rd quartile range (box), values extending to 1.5 times the interquartile range (whiskers), and outliers (dots). Only expressed genes were included for this analysis. Red asterisk indicates statistically significant difference between the two median values under comparison, p <2.2e-16 (student's t-test, two-sided). Source data are provided as Source Data file.

Supplementary References

1. Kumar, S., Stecher, G. & Tamura, K. MEGA7: Molecular Evolutionary Genetics Analysis Version 7.0 for bigger datasets. *Mol Biol Evol* **33**, 1870-4 (2016).
2. Xiao, J. *et al.* Cis and trans determinants of epigenetic silencing by Polycomb repressive complex 2 in Arabidopsis. *Nat Genet* **49**, 1546-1552 (2017).
