# Supplementary material for: The characterization of Mediator 12 and 13 as conditional positive gene regulators in Arabidopsis
Source: Nat Commun. 2020 Jun 3;11:2798. doi: 10.1038/s41467-020-16651-5 (PMC7271234; doi:10.1038/s41467-020-16651-5)
Supplement: Supplementary file 2 — Reporting Summary [file 41467_2020_16651_MOESM2_ESM.pdf]

nature research

Corresponding author(s): Steven E. Jacobsen

Last updated by author(s): Feb 1, 2020

Reporting Summary

Nature Research wishes to improve the reproducibility of the work that we publish. This form provides structure for consistency and transparency in reporting. For further information on Nature Research policies, see Authors & Referees and the Editorial Policy Checklist .

Statistics

For all statistical analyses, confirm that the following items are present in the figure legend, table legend, main text, or Methods section.

n/a	Confirmed	
☐	☒	The exact sample size (n) for each experimental group/condition, given as a discrete number and unit of measurement
☒	☐	A statement on whether measurements were taken from distinct samples or whether the same sample was measured repeatedly
☐	☒	The statistical test(s) used AND whether they are one- or two-sided<br>*Only common tests should be described solely by name; describe more complex techniques in the Methods section.*
☒	☐	A description of all covariates tested
☒	☐	A description of any assumptions or corrections, such as tests of normality and adjustment for multiple comparisons
☐	☒	A full description of the statistical parameters including central tendency (e.g. means) or other basic estimates (e.g. regression coefficient) AND variation (e.g. standard deviation) or associated estimates of uncertainty (e.g. confidence intervals)
☐	☒	For null hypothesis testing, the test statistic (e.g. F, t, r) with confidence intervals, effect sizes, degrees of freedom and P value noted<br>*Give P values as exact values whenever suitable.*
☒	☐	For Bayesian analysis, information on the choice of priors and Markov chain Monte Carlo settings
☒	☐	For hierarchical and complex designs, identification of the appropriate level for tests and full reporting of outcomes
☒	☐	Estimates of effect sizes (e.g. Cohen's d, Pearson's r), indicating how they were calculated

Our web collection on statistics for biologists contains articles on many of the points above.

Software and code

Policy information about availability of computer code

Data collection	No software was used during data collection.
Data analysis	Mapping by sequencing were performed using SHOREmap(v2.0) with default settings;<br><br>RNA-seq reads were quality filtered using trim_galore (v0.5.0 Babraham Bioinformatics, http://www.bioinformatics.babraham.ac.uk/projects/trim_galore/). The filtered reads were aligned to Arabidopsis TAIR10 reference genome using STAR(v2.7.0c). A java program MarkDuplicates.jar from the picard-tools suite was used to remove PCR duplicates from the resulting BAM files. We then obtained read counts of all genes and TEs according to Araport11 annotation file in the .gtf format using htseq-count v.0.6.1.p1; samtools(v1.2) was used to process the resulting BAM files.<br><br>Raw bisulfite sequencing reads were mapped to TAIR10 reference genome using BSMAP (v2.90). The enrichments of DNA methylation over selected regions were plotted using the ViewBS MethOverRegion function of the ViewBS package (v0.1.9);<br><br>The mapping of ChIP-seq reads were performed same as described for RNA-seq. To define ChIP-seq peaks, BAM files were loaded into macs2 (v2.1.2) callpeak function. To visualize the enrichments of different histone modifications over selected regions, we used ngsplot (v2.61).

For manuscripts utilizing custom algorithms or software that are central to the research but not yet described in published literature, software must be made available to editors/reviewers. We strongly encourage code deposition in a community repository (e.g. GitHub). See the Nature Research guidelines for submitting code & software for further information.

Data

Policy information about availability of data

All manuscripts must include a data availability statement. This statement should provide the following information, where applicable:

- Accession codes, unique identifiers, or web links for publicly available datasets
- A list of figures that have associated raw data
- A description of any restrictions on data availability

Both raw and processed sequencing reads were uploaded to GEO accession: GSE143835. Go to https://www.ncbi.nlm.nih.gov/geo/query/acc.cgi?acc=GSE143835. Token for reviewers: yvoromeolnsxdmd. Arabidopsis TAIR10 reference genome sequence was downloaded from the following site: https://www.arabidopsis.org/download/index-auto.jsp?dir=%2Fdownload_files%2FGenes%2FTAIR10_genome_release%2FTAIR10_chromosome_files

Field-specific reporting

Please select the one below that is the best fit for your research. If you are not sure, read the appropriate sections before making your selection.

☒ Life sciences ☐ Behavioural & social sciences ☐ Ecological, evolutionary & environmental sciences

For a reference copy of the document with all sections, see nature.com/documents/nr-reporting-summary-flat.pdf

Life sciences study design

All studies must disclose on these points even when the disclosure is negative.

Sample size

We collected and combined 5 Arabidopsis seedlings for each RNA extraction (one biological replicate), followed by library preparation and high-throughput sequencing. 3-5 biological replicates were analyzed for each genetic background. The sample size was determined empirically and followed the standard practice of the field. 3-5 biological replicates are enough to generate appropriate amount of differentially regulated genes of statistical significance.We collected and combined 5 Arabidopsis seedlings for each RNA extraction (one biological replicate), followed by reverse transcription and realtime-PCR. 5 biological replicates were analyzed for each genetic backgrounds. Each biological replicate is consisted of 3 technical replicates (PCR replicates). The sample size was determined based on the fact that the positive and negative control can be safely distinguished from each other, so that any gene products that is differentially accumulated comparing to the control can be defined with statistical significance.

10g of Arabidopsis seedlings (300-500 individuals) were collected and combined together (one biological replicate) for ChIP-seq experiments. Two biological replicates were used for ChIP-seq analysis.10g of Arabidopsis seedlings (300-500 individuals) were collected and combined together (one biological replicate) for IP-Mass Spectrometry experiments. Two biological replicates were used.Plant flowering time was calculated from 20-40 adult Arabidopsis plants for each genetic background.

The GFP phenotype of 20-30 individual plants were examined for each genetic background. Pictures of 5 representative plants were taken.

No data were excluded from the analysis.

Data exclusions

Replication

Attempts for data replication were successful. Specifically:

We collected and combined 5 Arabidopsis seedlings for each RNA extraction (one biological replicate), followed by library preparation and high-throughput sequencing. 3-5 biological replicates were analyzed for each genetic background.

We collected and combined 5 Arabidopsis seedlings for each RNA extraction (one biological replicate), followed by reverse transcription and realtime-PCR. 5 biological replicates were analyzed for each genetic backgrounds. Each biological replicate is consisted of 3 technical replicates (PCR replicates).

10g Arabidopsis seedlings (300-500 individuals) were collected and combined together (one biological replicate) for ChIP-seq experiments. Two biological replicates were used for ChIP-seq analysis.

10g Arabidopsis seedlings (300-500 individuals) were collected and combined together (one biological replicate) for IP-Mass Spectrometry experiments. Two biological replicates were used.

Plant flowering time was calculated from 20-40 adult Arabidopsis plants for each genetic background.

The GFP phenotype of 20-30 individual plants were examined for each genetic background. Pictures of 5 representative plants were taken.

Randomization

Plant samples under comparison (flowering time, GFP expression, RNA-seq, etc.) were grown at the same time under the same condition. The exact position of each plant individual was always randomized, so that small environmental variations were mitigated as much as possible.

Blinding was not possible during this study, because specific control has to be designated for data normalization (realtime-PCR, RNA-seq, ChIP-seq).

Blinding

Reporting for specific materials, systems and methods

We require information from authors about some types of materials, experimental systems and methods used in many studies. Here, indicate whether each material, system or method listed is relevant to your study. If you are not sure if a list item applies to your research, read the appropriate section before selecting a response.

Materials & experimental systems

n/a	Involved in the study
☐	☒ Antibodies
☒	☐ Eukaryotic cell lines
☒	☐ Palaeontology
☒	☐ Animals and other organisms
☒	☐ Human research participants
☒	☐ Clinical data

Methods

n/a	Involved in the study
☐	☒ ChIP-seq
☒	☐ Flow cytometry
☒	☐ MRI-based neuroimaging

Antibodies

Antibodies used

5 µl anti-H3 antibody (Abcam #1791), 5 µl of anti-H3K4m3 antibody (Millipore 04-745), 5 µl of anti-H3K9me2 antibody (Abcam #1220), 10 µl of anti-H3K27me3 antibody (Millipore 07-449), 5 µl of anti-H3K36me3 antibody (Abcam #9050), 5 µl of anti-H3PanAc antibody (Active Motif #39139), and 5 µl of anti-FLAG antibody (Sigma-A8592-1MG) were used for each ChIP sample. All antibodies used in this study were undiluted.

Validation

Please refer to the provided link for product validation:
Ab1791 (https://www.abcam.com/histone-h3-antibody-nuclear-loading-control-and-chip-grade-ab1791.html);

Millipore 04-745 (https://www.merckmillipore.com/CN/zh/product/Anti-trimethyl-Histone-H3-Lys4-Antibody-clone-MC315-rabbit-monoclonal,MM_NF-04-745?ReferrerURL=https%3A%2F%2Fwww.google.com%2F#anchor_COA);

Ab1220 (https://www.abcam.com/histone-h3-di-methyl-k9-antibody-mabcam-1220-chip-grade-ab1220.html?productWallTab=ShowAll);

Millipore 07-449 (https://www.merckmillipore.com/CN/zh/product/Anti-trimethyl-Histone-H3-Lys27-Antibody,MM_NF-07-449?ReferrerURL=https%3A%2F%2Fwww.google.com%2F);

Ab9050 (https://www.abcam.com/histone-h3-tri-methyl-k36-antibody-chip-grade-ab9050.html);

Active Motif 39139 (https://www.activemotif.com/catalog/details/39139/histone-h3ac-pan-acetyl-antibody-pab-1);

Sigma-A8592 (https://www.sigmaaldrich.com/catalog/product/sigma/a8592?lang=zh&region=CN).

Animals and other organisms

Policy information about studies involving animals; ARRIVE guidelines recommended for reporting animal research

Laboratory animals	The study did not involve laboratory animals.
Wild animals	The study did not involve wild animals.
Field-collected samples	The study did not involve samples collected from the fields.
Ethics oversight	No ethical approval was required. The study was conducted exclusively on plant model species Arabidopsis thaliana.

Note that full information on the approval of the study protocol must also be provided in the manuscript.

ChIP-seq

Data deposition

☒ Confirm that both raw and final processed data have been deposited in a public database such as GEO.

☒ Confirm that you have deposited or provided access to graph files (e.g. BED files) for the called peaks.

Data access links
May remain private before publication.

Both raw and processed sequencing reads were uploaded to GEO accession: GSE143835. Go to https://www.ncbi.nlm.nih.gov/geo/query/acc.cgi?acc=GSE143835. Token for reviewers: yvoromeolnsxdmd

Files in database submission

Col_H3.bw
Col_H3K4me3.bw
Col_H3K9me2.bw
Col_H3K27me3.bw
Col_H3K36me3.bw
Col_H3PanAc.bw
Col_input.bw
ddc_H3_rep1.bw
ddc_H3_rep2.bw
ddc_H3K4me3_rep1.bw
ddc_H3K4me3_rep2.bw
ddc_H3K9me2_rep1.bw
ddc_H3K9me2_rep2.bw
ddc_H3K27me3_rep1.bw
ddc_H3K27me3_rep2.bw
ddc_H3K36me3_rep1.bw
ddc_H3K36me3_rep2.bw
ddc_H3PanAc_rep1.bw
ddc_H3PanAc_rep2.bw
ddc_input_rep1.bw
MED12-FLAG_col_control.bw
MED12-FLAG_Dark_rep1_input.bw
MED12-FLAG_Dark_rep1.bw
MED12-FLAG_Dark_rep2.bw
MED12-FLAG_light_rep1_input.bw
MED12-FLAG_light_rep1.bw
MED12-FLAG_light_rep2.bw
MED12-FLAG_rep1_input.bw
MED12-FLAG_rep1.bw
MED12-FLAG_rep2_input.bw
MED12-FLAG_rep2.bw
morc1_H3_rep1.bw
morc1_H3_rep2.bw
morc1_H3K4me3_rep1.bw
morc1_H3K4me3_rep2.bw
morc1_H3K9me2_rep1.bw
morc1_H3K9me2_rep2.bw
morc1_H3K27me3_rep1.bw
morc1_H3K27me3_rep2.bw
morc1_H3K36me3_rep1.bw
morc1_H3K36me3_rep2.bw
morc1_H3PanAc_rep1.bw
morc1_H3PanAc_rep2.bw
morc1_input_rep1.bw
SDCGFP_col_H3.bw
SDCGFP_col_H3K4me3.bw
SDCGFP_col_H3K9me2.bw
SDCGFP_col_H3K27me3.bw
SDCGFP_col_H3K36me3.bw
SDCGFP_col_H3PanAc.bw
SDCGFP_col_input.bw
col_H3.fastq.gz
col_H3K4me3.fastq.gz
col_H3K9me2.fastq.gz
col_H3K27me3.fastq.gz
col_H3K36me3.fastq.gz
col_H3PanAc.fastq.gz
col_input.fastq.gz
ddc_H3_rep1.fastq.gz
ddc_H3_rep2.fastq.gz
ddc_H3K4me3_rep1.fastq.gz
ddc_H3K4me3_rep2.fastq.gz
ddc_H3K9me2_rep1.fastq.gz
ddc_H3K9me2_rep2.fastq.gz
ddc_H3K27me3_rep1.fastq.gz
ddc_H3K27me3_rep2.fastq.gz
ddc_H3K36me3_rep1.fastq.gz

ddc_H3K36me3_rep2.fastq.gz
ddc_H3PanAc_rep1.fastq.gz
ddc_H3PanAc_rep2.fastq.gz
ddc_input_rep1.fastq.gz
MED12-FLAG_col_control.fastq.gz
MED12-FLAG_dark_rep1_input.fastq.gz
MED12-FLAG_dark_rep1.fastq.gz
MED12-FLAG_dark_rep2.fastq.gz
MED12-FLAG_light_rep1_input.fastq.gz
MED12-FLAG_light_rep1.fastq.gz
MED12-FLAG_light_rep2.fastq.gz
MED12-FLAG_rep1_input.fastq.gz
MED12-FLAG_rep1.fastq.gz
MED12-FLAG_rep2_input.fastq.gz
MED12-FLAG_rep2.fastq.gz
morc1_H3_rep1.fastq.gz
morc1_H3_rep2.fastq.gz
morc1_H3K4me3_rep1.fastq.gz
morc1_H3K4me3_rep2.fastq.gz
morc1_H3K9me2_rep1.fastq.gz
morc1_H3K9me2_rep2.fastq.gz
morc1_H3K27me3_rep1.fastq.gz
morc1_H3K27me3_rep2.fastq.gz
morc1_H3K36me3_rep1.fastq.gz
morc1_H3K36me3_rep2.fastq.gz
morc1_H3PanAc_rep1.fastq.gz
morc1_H3PanAc_rep2.fastq.gz
morc1_input_rep1.fastq.gz
SDCGFP_col_H3.fastq.gz
SDCGFP_col_H3K4me3.fastq.gz
SDCGFP_col_H3K9me2.fastq.gz
SDCGFP_col_H3K27me3.fastq.gz
SDCGFP_col_H3K36me3.fastq.gz
SDCGFP_col_H3PanAc.fastq.gz
SDCGFP_col_input.fastq.gz

Genome browser session
(e.g. UCSC)

Bigwig files were uploaded to GEO accession: GSE143835. For data visualization please download the bigwig files at https://www.ncbi.nlm.nih.gov/geo/query/acc.cgi?acc=GSE143835, and visualize using IGV. Token for reviewers: yvoromeolnsxdmd

Methodology

Replicates

10g Arabidopsis seedlings (300-500 individuals) were collected and combined together (one biological replicate) for ChIP-seq experiments. Two biological replicates were used for ChIP-seq analysis.

Sequencing depth

MED12-FLAG_rep1, total number of reads 19,018,936, uniquely mapped reads 12,906,782, single-end 50bp.
MED12-FLAG_rep2, total number of reads 19,147,073, uniquely mapped reads 11,534,223, single-end 50bp.
MED12-FLAG_col_control, total number of reads 19,895,406, uniquely mapped reads 9,209,423, single-end 50bp.
Histone ChIP-seq data are of similar sequencing depth.

Antibodies

5 µl anti-H3 antibody (Abcam #1791), 5 µl of anti-H3K4m3 antibody (Millipore 04-745), 5 µl of anti-H3K9me2 antibody (Abcam #1220), 10 µl of anti-H3K27me3 antibody (Millipore 07-449), 5 µl of anti-H3K36me3 antibody (Abcam #9050), 5 µl of anti-H3PanAc antibody (Active Motif #39139), and 5 µl of anti-FLAG antibody (Sigma-A8592-1MG) were used for each ChIP sample.

Peak calling parameters

To define ChIP-seq peaks, BAM files were loaded into macs2 (v2.1.2) callpeak function with the following parameters: -f BAM -nomodel -g 1.3e8 -B -q 0.01. Peaks obtained from the above procedures were further filtered using the cut-off value -log10q-value >30.

H3K4me3, H3K9me2, H3K27me3, H3K36me3 and H3PanAc ChIP-seq peaks were called using H3 ChIP-seq as normalization control. Only H3 ChIP-seq data of the same genetic backgrounds were used for normalization.

MED12 ChIP-seq peaks were called by normalizing MED12-FLAG rep1 and MED12-FLAG rep2 to MED12-FLAG_col_control.

Data quality

2449 and 3127 MED12 ChIP-seq peaks were called from biological replicate 1 and 2 (FDR>0.01), respectively. Among them, 1072 and 1302 peaks show >5fold enrichments.
Peaks obtained from the initial analysis were further filtered using the cut-off value -log10q-value >30 for downstream analysis.

Software

ChIP-seq reads were quality filtered using trim_galore (Babraham Bioinformatics, http://www.bioinformatics.babraham.ac.uk/projects/trim_galore/). The filtered reads were aligned to Arabidopsis TAIR10 reference genome using STAR. A java program MarkDuplicates.jar from the picard-tools suite was used to remove PCR duplicates from the resulting BAM files.

To define ChIP-seq peaks, BAM files were loaded into macs2 (v2.1.2) callpeak function. To visualize the enrichments of different histone modifications over selected regions, we used ngsplot (v2.61).
