# Supplementary material for: The characterization of Mediator 12 and 13 as conditional positive gene regulators in Arabidopsis
Source: Nat Commun. 2020 Jun 3;11:2798. doi: 10.1038/s41467-020-16651-5 (PMC7271234; doi:10.1038/s41467-020-16651-5)
Supplement: Supplementary file 3 — Description of Additional Supplementary Files [file 41467_2020_16651_MOESM3_ESM.pdf]

Description of Additional Supplementary Files

File name: Supplementary Dataset 1
Description: RNAseq output
Contains the raw DEseq2 outputs for all RNAseq samples analyzed in this study.

File name: Supplementary Dataset 2
Description: MED12 MED13 DEG and Control
Contains the lists of genes that are differentially regulated in med12 and med13 mutants, and corresponding control gene sets used in histone mark comparison.

File name: Supplementary Dataset 3
Description: MED12 ChIPseq peaks
Contains the MACS2 defined MED12 ChIPseq peak region and MED12 fold enrichments for two MED12 ChIPseq replicates.

File name: Supplementary Dataset 4
Description: Gene expression tiers
Contains the lists of all Arabidopsis genes divided into 5 subgroups based on their expression levels in the wild type plants.

File name: Supplementary Dataset 5
Description: MED12 interacting gene IDs
Contains the lists of strongly MED12-interacting genes based on MED12 ChIPseq experiments.

File name: Supplementary Dataset 6
Description: MED12-interacting genes in different clusters
Contains the lists of MED12-interacting genes subdivided into groups based on their histone modification signatures.

File name: Supplementary Dataset 7
Description: light inducible gene IDs
Contains the lists of genes whose expression are significantly upregulated in the wild type plants upon light induction.

File name: Supplementary Dataset 8
Description: MED12 ChIP-seq peaks in dark and light conditions
Contains the raw ChIPseq results (MACS2 output) for two independent MED12-complementing lines in the dark- and light-treated conditions.

File name: Supplementary Dataset 9
Description: MED12-interacting genes in dark and light conditions
Contains the lists of MED12-interacting genes resulting from the ChIPseq analysis for two independent MED12-complementing lines in the dark and light treated conditions

File name: Supplementary Dataset 10
Description: MED12 IP-MS
Contains the lists of MED12-interacting proteins based on IP-MS results from two independent MED12-complementing lines.

File name: Supplementary Dataset 11
Description: Oligos used in this study
Contains the sequences information for all oligos used in this study.
